# Where did you come from, where did you go: Refining metagenomic analysis tools for horizontal gene transfer characterisation

**DOI:** 10.1371/journal.pcbi.1007208

**Published:** 2019-07-23

**Authors:** Enrico Seiler, Kathrin Trappe, Bernhard Y. Renard

**Affiliations:** 1 Bioinformatics Unit (MF1), Department for Methods Development and Research Infrastructure, Robert Koch Institute, Berlin, Germany; 2 Efficient Algorithms for Omics Data, Max Planck Institute for Molecular Genetics, and Algorithmic Bioinformatics, Institute for Bioinformatics, Freie Universität Berlin, Berlin, Germany; University College London, UNITED KINGDOM

## Abstract

Horizontal gene transfer (HGT) has changed the way we regard evolution. Instead of waiting for the next generation to establish new traits, especially bacteria are able to take a shortcut via HGT that enables them to pass on genes from one individual to another, even across species boundaries. The tool Daisy offers the first HGT detection approach based on read mapping that provides complementary evidence compared to existing methods. However, Daisy relies on the acceptor and donor organism involved in the HGT being known. We introduce DaisyGPS, a mapping-based pipeline that is able to identify acceptor and donor reference candidates of an HGT event based on sequencing reads. Acceptor and donor identification is akin to species identification in metagenomic samples based on sequencing reads, a problem addressed by metagenomic profiling tools. However, acceptor and donor references have certain properties such that these methods cannot be directly applied. DaisyGPS uses MicrobeGPS, a metagenomic profiling tool tailored towards estimating the genomic distance between organisms in the sample and the reference database. We enhance the underlying scoring system of MicrobeGPS to account for the sequence patterns in terms of mapping coverage of an acceptor and donor involved in an HGT event, and report a ranked list of reference candidates. These candidates can then be further evaluated by tools like Daisy to establish HGT regions. We successfully validated our approach on both simulated and real data, and show its benefits in an investigation of an outbreak involving Methicillin-resistant Staphylococcus aureus data.

## Author summary

Evolution is traditionally viewed as a process where changes are only vertically inherited from parent to offspring across generations. Many principles such as phylogenetic trees and even the “tree of life” are based on that doctrine. The concept of horizontal gene transfer changed the way we regard evolution completely. Horizontal gene transfer is the movement of genetic information between distantly related organisms of the same generation. Genome sequencing not only provided further evidence complementing experimental evidence but also shed light onto the frequency and prominence of this concept. Especially the rapid spread of antimicrobial resistance genes is a prominent example for the impact that horizontal gene transfer can have for public health. Next generation sequencing brought means for quick and relatively cheap analysis of even complex metagenomic samples where horizontal gene transfer is bound to happen frequently. Methods to directly detect and characterise horizontal gene transfer from such sequencing data, however, are still lacking. We here provide a method to identify organisms potentially involved in horizontal gene transfer events to be used in downstream analysis that enables a characterisation of a horizontal gene transfer event in terms of impact and prevalence.

## Introduction

**Fig 1:**
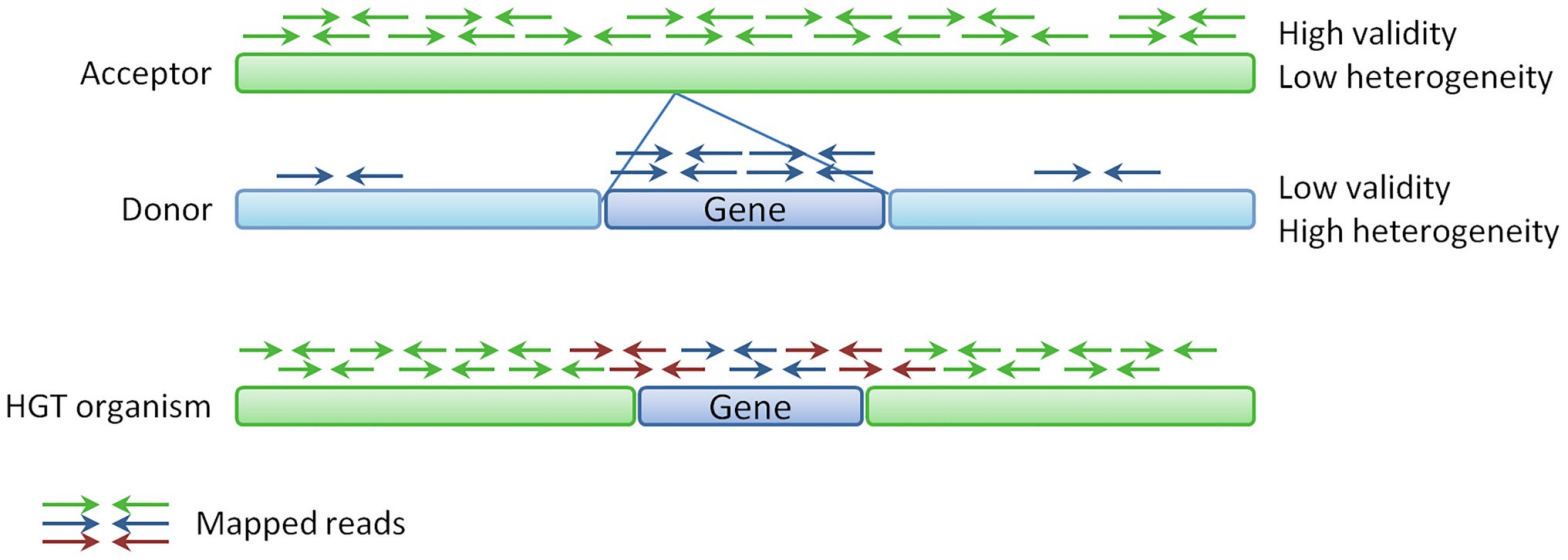
HGT overview and evidence. The sequence of an HGT organism consists mainly of the sequence of the acceptor genome (green), and only the transferred part (blue gene) is represented by the donor genome. Hence, reads from the HGT organism should mainly map homogeneously to the acceptor (green arrows), only few reads should map locally to the donor (blue arrows), and some read pairs (red arrows) will span the boundary between the green parts from the acceptor and the blue part from the donor. These mapping patterns can be represented by scores based on the mapping coverage profile. An acceptor with a homogeneous coverage has a high validity score and a low heterogeneity score, a donor has opposite score ranges (low validity and high heterogeneity). Based on these scores, the DaisyGPS *acceptor*-*score* is ∈ [0, 1] and *donor*-*score* is ∈ [−1, 0).

For a long time, evolution in terms of gene transfer was thought to happen only along the tree of life, i.e. from parent to offspring generation. The discovery of horizontal gene transfer (HGT) [1–4] has revolutionised this dogma, and revealed the mechanism that enables bacteria to quickly adapt to environmental pressure [5–7]. Via HGT, bacteria can directly transfer one or multiple genes from one individual to another across species boundaries. The known and prominent mechanisms of HGT are transformation (uptake of nascent DNA from the environment), conjugation (direct transfer from cell to cell), and transduction (transfer via bacteriophages) [7]. In all cases, a piece of DNA sequence is—directly or indirectly—transferred from the so called donor organism to the acceptor organism and integrated into the genome (see also Fig 1).

Especially conjugation and transduction facilitate the transfer of pathogenicity islands and mobile genetic elements involving antimicrobial resistance (AMR) genes [8–10]. Today, we are facing the rise of so called “superbugs” [10, 11] as a result of bacterial adaptation and gain of resistance to antibiotic treatment, showing the need for methods to identify, characterise and trace HGT events.

The discrepancy between vertical, phylogenetic evolution and evidence for horizontal exchange and evolution across branches of a phylogenetic tree inspired existing genome-based HGT methods. For a fixed set of species and a potential horizontally transferred gene, these methods detect HGT events by looking at inconsistencies between the gene tree and a phylogenetic tree built for the set of species [12]. As a prerequisite, a candidate gene for which to run the calculation and comparison has to be known. Sequence content based methods aim to identify genes of foreign origin in a given genome by exploiting sequence pattern such as *k-mer* frequencies or GC content which vary between different species [13], [14]. All methods are based on an assembled genomes, meaning they are also prone to the problems of misassemblies. Although AMRs are a prominent example for horizontally transferred genes, methods to directly identify antimicrobial resistance (AMR) genes do not necessarily connect the presence of an AMR gene to an HGT event (e.g., KmerResistance [15]).

In previous work, we developed an approach that aims to call HGT events directly from next-generation sequencing (NGS) data [16] in a tool called Daisy. Instead of focusing on the sequence content or rather inconsistencies in the sequence content of the organism that acquired genes through HGT, Daisy examines the origin of the transfer, namely the prespecified acceptor and the donor organisms, and directly maps the NGS reads to these references. By facilitating structural variant detection methods, we can thereby identify the transferred region from the donor and the insertion site within the acceptor. A prerequisite for Daisy is therefore that both acceptor and donor references are known. This, however, is not always the case, and hence requires methods that are able to infer acceptor and donor reference candidates from the NGS reads of the organism assumed to be the result of an HGT event. Such methods are not yet available.

However, the problem of acceptor and donor identification directly from NGS data is akin to the problem tackled by metagenomic profiling studies that aim to unravel metagenomic samples. Here, so called metagenomic classification approaches aim at identifying all organisms present in a sample by directly analysing sequencing data with a complex mixture of various organisms [17]. While in this classical scenario all reads of a single organism in the sample can theoretically be assigned to one reference organism during identification, this is not the case for an organism that carries foreign genes acquired via HGT. Most reads will be assigned to the acceptor genome but only a fraction can map to the donor genome (see mapped reads in Fig 1). Hence, we have to account for this two mapping properties of the reads during analysis. Another requirement is the resolution of classification on strain level, if possible, since two strains of the same species can already significantly differ in their sequence content.

Metagenomic classification approaches follow either a taxonomy dependent or taxonomy independent approach [18, 19]. The general procedure for both approaches is to assign sequencing reads stemming from the same organism in the sample into the same group, a process also referred to as binning. Taxonomic dependent binning approaches assign the reads to specific taxonomic groups, and hereby infer the presence of these taxa in the sample. These methods either also make use of sequence composition patterns, e.g., Kraken [20], or they determine mapping-based sequence similarities for the read assignment, e.g., MEGAN [21], Clinical PathoScope [22] or DUDes [23]. Both approaches will most likely identify the acceptor reference of an HGT organism due to the homogeneous coverage and comparatively high number of reads. The drawback of all read assignment approaches is the limitation in the presence of mobile genetic elements, e.g., integrated via HGT or of hitherto unknown—or unsequenced—organisms in the sample. Reads belonging to these genes or unknown organisms are either assigned to a similar but incorrect taxa or not assigned at all, leading to wrong identifications and biases in abundance estimation. To ensure robustness, many approaches deliberately discard taxonomic candidates with only low and local coverage. Hence these approaches will likely discard any donor candidate references. Composition-based methods such as Kraken would also perform poorly pinpointing the correct donor based on evidence of only few reads given the fairly large number of usually detected species.

In our group, we developed MicrobeGPS [24], a metagenomics approach that accounts for sequences not yet present in the database. Instead of reporting fixed taxa with assigned reads, MicrobeGPS in turn uses the candidate taxa to describe the organisms in the sample in terms of a genomic distance measure. That is, it uses available references to model the composition of the organisms present in the sample in terms of coverage profiles and continuity, instead of directly assigning reference organisms to characterize the sample. If the organism in the sample is present in the database and covered homogeneously then the distance approximates to zero. If not, MicrobeGPS identifies the closest relatives by positioning the organism among references with the lowest genomic distance. Hence, the tool considers scores and metrics that reflect a donor-like, in-homogeneous coverage but filters out false positive candidates with inhomogeneous coverage for the purpose of species assignment. From the perspective of HGT detection, these may be highly relevant and should not be excluded.

Here we present DaisyGPS, a pipeline building on concepts of MicrobeGPS and tailored to the identification of acceptor and donor candidates from sequencing reads of an organism that may be involved in an HGT event. DaisyGPS uses genome distance metrics to define a score that allows the classification into acceptor and donor among the reported organisms. Owing to the properties of these scores, we still find the closest relatives of acceptor and donor in case these references are not present in the database. DaisyGPS further offers optional blacklists and a species filter to refine the search space for acceptor and donor candidates. DaisyGPS and Daisy are integrated into one pipeline called DaisySuite to offer a comprehensive HGT detection. We validate DaisySuite on a large-scale simulation where we show sensitivity and specificity of our approach and the robustness when applied to non-HGT samples. By simulating evolutionary distances, we demonstrate in another experiment that DaisySuite can detect HGTs in organisms that diverge from the original acceptor and donor. In addition, we used the simulated metagenomic data sets from the CAMI challenge [25] in combination with our simulated HGT reads to show that DaisySuite is able to detect HGTs in metagenomic samples. On a real data set from an Methicillin-resistant Staphylococcus aureus (MRSA) outbreak, we demonstrate the ability of the DaisySuite to distinguish between the outbreak associated and unassociated samples in terms of sequenced content potentially acquired through HGT events.

## Materials and methods

**Fig 2:**
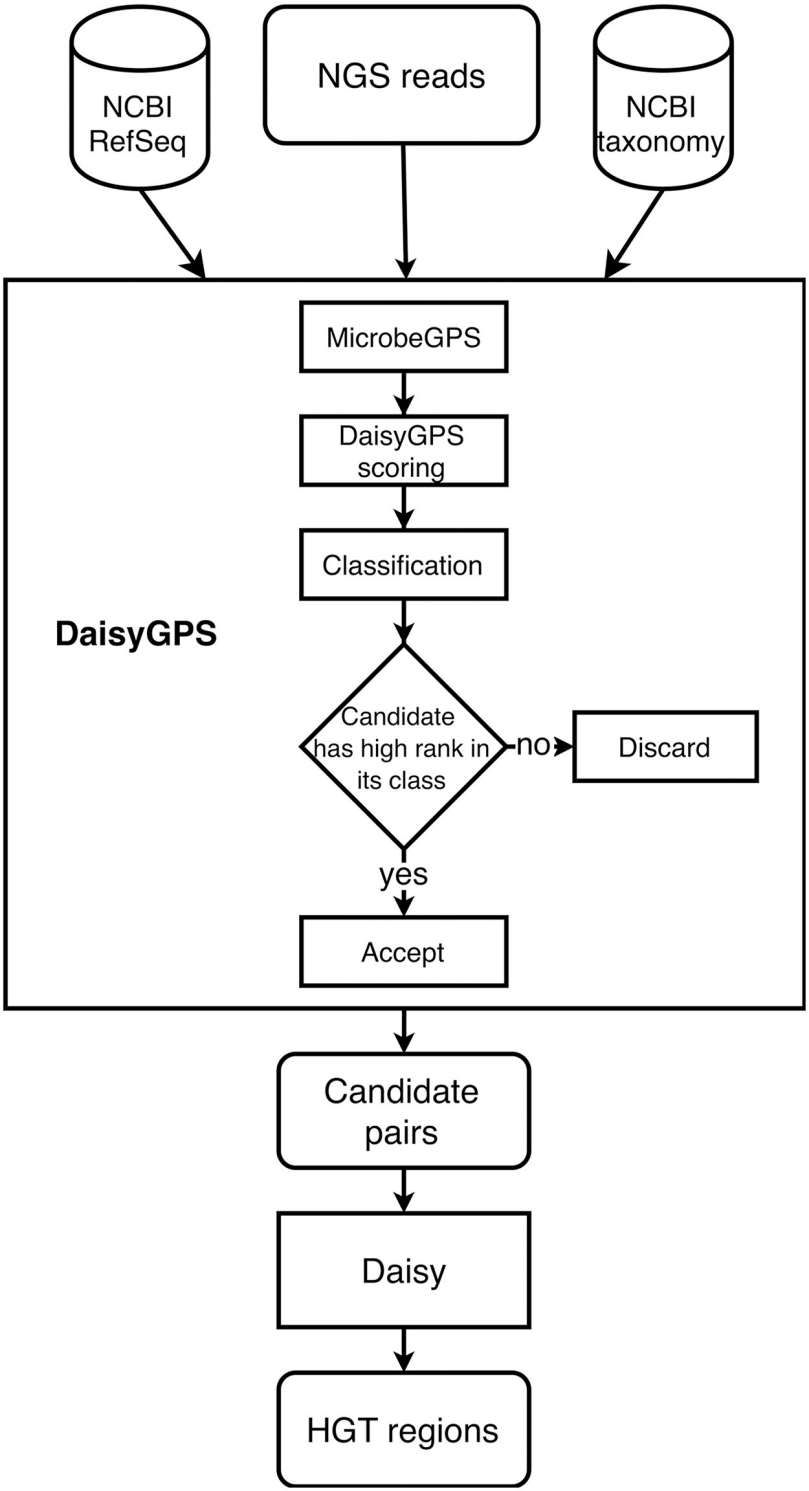
Workflow of DaisySuite. The input NGS reads are first processed by DaisyGPS. The reads are mapped to the NCBI RefSeq and then analysed by MicrobeGPS which also incorporates taxonomic information acquired through the NCBI taxonomy database. Based on that, DaisyGPS calculates two scores for acceptor and donor classification (see Methods part). Depending on these scores, the highest-ranked candidates are selected as suitable acceptor and donor candidates. Daisy then uses these candidates to identify HGT region candidates.

The problem of mapping-based HGT detection from NGS data is twofold: First, the acceptor reference (organism that receives genetic information) and donor reference (organism that the information is transferred from) that are involved in the HGT event have to be identified. In the following, we refer to the organism that derived from the acceptor and acquired genes from the donor in an HGT event as an *HGT organism*. Based on that, the precise HGT region from the donor and its insertion site within the acceptor can be characterised. We presented a method to solve the second task in [16]. Here, we propose the tool DaisyGPS (see also Fig 2) with the objective to identify possible acceptor and donor genome candidates given reads of a—pure or metagenomic—sample containing a potential HGT organism. We provide Daisy and DaisyGPS in an integrated pipeline that we call DaisySuite. DaisySuite is publically available at https://gitlab.com/rki_bioinformatics/DaisySuite, an extended documentation can be found at https://daisysuite.readthedocs.io/en/latest/index.html.

The genome of the HGT organism consists mainly of the acceptor genome (see Fig 1). When the reads of the HGT organism are mapped against the acceptor reference, most reads should map properly. Therefore a high and continuous mapping coverage pattern of the acceptor genome can be expected. In contrast to that, only a small part of the donor genome is present within the genome of the HGT organism, hence only a small fraction of the reads should map against the donor reference and then only within a zoned part (i.e. the part that has been transferred). This results in a discontinuous mapping coverage pattern where only a small part of the reference shows a high mapping coverage (see Fig 1).

In a first step, we need to define metrics that represent the expectations we have, i.e. how much of the genome is covered by reads (mapping coverage) and how uniformly these reads are distributed across the genome (discontinuous vs. continuous patterns). Given only the reads of the HGT organism, the acceptor and donor candidate identification problem is similar to aspects of metagenomic profiling. A standard problem in metagenomics is the identification of organisms in a sample using a read data set of this sample. At first glance, it may appear that the methods designed to solve this problem can also be applied to our identification objective, i.e. we have the read data set of the HGT organism and we are looking for two organisms (acceptor and donor) that are in the sample. However, because the HGT organism consists mainly of the acceptor genome, such an approach works only well for the identification of the acceptor. For the donor, additional information is needed to guarantee a reliable identification because references with only local or discontinuous coverage are usually dismissed by the profiler. We use the metagenomic profiling tool MicrobeGPS to obtain a coverage profile of our given HGT organism from mapping coverage metrics. MicrobeGPS fits our requirements as it can be configured to not filter any organisms and reports additional metrics that we use to represent acceptor and donor attributes. We evaluate the gathered metrics and establish a score that reflects our defined acceptor or donor coverage properties. The candidates are ranked by this score and a list of acceptor and donor candidates is generated. These acceptor and donor candidates can then be further analysed with tools such as Daisy.

### DaisyGPS scores

For the purpose of HGT detection, we aim to define a scoring that reflects the mapping coverage properties of the acceptor and donor references: The acceptor has a continuous, homogeneous coverage over the complete length of the genome. The donor has a local, but still homogeneous coverage in the area where the transferred genes are originated but should have nearly no coverage at all otherwise. The score should further allow a clear distinction between acceptor and donor candidates and provide a meaningful ranking according to the likelihood of being the most suitable candidate.

As a basis for our scoring, we use the *Genome Dataset Validity* defined in [26] and *homogeneity* metric defined in [24]. The Genome Dataset Validity, or short validity, describes the fraction of the reference genome for which there is read evidence. In contrast, the homogeneity reflects how evenly the reads are distributed. Both have a range ∈ [0, 1]. The validity is defined such that a genome that is covered—either low or high—over the full length has a high validity (≈ 1). The validity can be interpreted as a measure of sequence similarity between the sequenced sample and a reference genome. Analogous to the homogeneity metric, we define a *heterogeneity* metric based on the Kolmogorov-Smirnov test statistic defined in [24] such that an evenly covered genome has a low heterogeneity (≈ 0) and a genome with local, high coverage a high heterogeneity (≈ 1). Note that the heterogeneity is a vertical translation of the homogeneity defined in [24], i.e. *heterogeneity* = 1 − *homogeneity*.

An acceptor is a genome with a continuous, high coverage that then has a high validity (≈ 1) and a low heterogeneity (≈ 0) score whereas a distantly related donor genome with only local, discontinuous coverage has a low validity (≈ 0) and a high heterogeneity (≈ 1) score.

As can be seen above, both validity and heterogeneity are complementary for acceptors and donors, and hence the relation of both metrics infers the property of a candidate between being an acceptor or a donor candidate.

We define:

$$\begin{array}{ccc} score=validity-heterogeneity & \text{with} & score\in [-1,1] \end{array}$$ (1)

Acceptor candidates have a homogeneous coverage and hence high validity and low heterogeneity, i.e. *validity* > *heterogeneity*. Therefore, the value for a completely covered acceptor with uniform read distribution would approach +1. Likewise, the value for a donor that is only covered in a small region would approach −1. In addition to the coverage profile, there is a high evidence by sheer read numbers for acceptors:

$$\begin{array}{ccc} acceptor-score=w*score & \text{with} & w=\frac{\#mapped\;reads}{\#total\;reads} \end{array}$$ (2)

where *w* is the fraction of all mapped reads that mapped to the specific acceptor candidate. For the donor, however, the size of the transferred region is not known in advance. Hence, we do not expect a specific read number evidence and therefore omit the weighting and define

$$\begin{array}{c} donor-score=score \end{array}$$ (3)

Both *acceptor*-*score* and *donor*-*score* are determined for every candidate and they have a codomain of [-1, 1]. Hence, we classify the candidates with *acceptor*-*score* ≥ 0 as acceptor and rank them from highest to lowest score. Donor candidates have a high heterogeneity and low validity, i.e. *validity* < *heterogeneity*. Therefore, we classify candidates with *donor*-*score* < 0 as donor candidates and rank them from lowest to highest score.

There is a special case if acceptor and donor are very similar. Here, the donor might not express the attributes we are looking for. In particular, the donor might have a significant read number evidence arising from acceptor reads also mapping to the donor. These shared reads lead to more regions of the donor genome being covered (higher validity) and to a less local, more homogeneous coverage pattern across the donor genome (lower heterogeneity), hence *validity* ≈ *heterogeneity* and *donor*-*score* ≈ 0. For such an event to occur, the true acceptor itself must be covered well (and evenly) enough to exhibit the hallmarks of an acceptor. Given that the donor is highly similar to the acceptor, a prime example being *E. coli* and *Shigella*, the validity of the donor strongly increases while the heterogeneity still takes the highly covered parts originating from the transferred region into account, allowing a positive *donor*-*score*. In contrast to this, a negative *donor*-*score* may easily occur due to spurious reads mapping to a reference genome without high similarity to the acceptor. Hence, we introduce a third classification and classify candidates with a *donor*-*score* > 0 as acceptor-like donors and rank them from lowest to highest.

A user definable number of the highest ranked candidates of each class (default: two acceptors, three donors and two acceptor-like donors) is then used to report all possible acceptor-donor candidate pairs, i.e. the cartesian product acceptors × (donors ∩ acceptor-like donors). For all these pairs, a follow-up Daisy run is triggered.

### Candidate selection with blacklist filter (optional)

There are scenarios where it is necessary to exclude certain candidates from being reported. For example, in a reanalysis case, the assembled sequence from the sample reads might already been added to the reference set of your choice. For HGT detection from such reads, however, there is no information gain if DaisyGPS reports this entry as a suitable acceptor. Other examples include cases, where one can exclude certain species or taxa due to preanalysis information that nevertheless could be reported by DaisyGPS due to their high sequence similarity to the sampled organism or the presumed acceptor or donor candidates. To make the search for acceptor and donor candidates adaptable for such cases, DaisyGPS features the blacklisting of certain taxa. It is possible to exclude single taxa, a complete species taxon or a complete subtree below a specified taxon. For a default run, the filter is turned off.

### Candidate selection with species filter (optional)

DaisyGPS generally considers candidates on different taxonomic levels, e.g. species and strain level, and reports the candidate level with the best scores. Often the strain references contain additional sequences compared to the species level reference representative, and hence, the species reference will mostly have a homogeneous coverage that will then lead to a high acceptor score. Usually identification on species level is sufficient. There are however species such as, e.g., *E.coli*, where a high number of strains have been sequenced already and differ in their properties such as pathogenicity among the strains (e.g. *E.coli* K12 versus EHEC strain O157:H7). In these cases, a mere detection of the acceptor or donor on a species level might not be precise enough. For these situations, we implemented a species filter. If this filter is activated, only candidates below species level are reported. In case no candidate would be reported with an active species filter, the filter is disabled and the user informed that for further analysis also candidates on species level are used. For a default run, this filter is also turned off.

### Candidate selection with limited number of reports per species (optional)

By default, DaisyGPS reports multiple acceptor candidates within the same species, given that they have equally high scores. If such a candidate organism is within an overrepresented group of the database, e.g., *E. coli*, they are often also overrepresented in the reported candidates due to the high similarity between strains of the same species. In this case, it can be beneficial to allow a broader view over the possible candidates by restricting the number of reported species representatives. Another use case can emerge when a priori knowledge about a donor exists and, optionally in combination with other filters, a more verbose overview of suitable species is prefered. For such occasions, we implemented a filter that allows to specify how many candidates per species are reported. We recommend to use this filter for metagenomic samples to reflect the high diversity of the sample among the acceptor and donor candidates.

### Daisy integration and integration with Snakemake

Snakemake is a common workflow management system [27] which we used to implement the different steps of DaisyGPS. We generated the alignment file required for MicrobeGPS by mapping the reads of the HGT organism against the NCBI RefSeq (complete RefSeq, no plasmids, downloaded March 15th 2017) [28] using Yara [29, 30] in *all-mapper* mode, i.e. all suitable hits are reported for each hit. To ensure compatibility, we reimplemented the Daisy workflow in Snakemake as well, and integrated both into a combined suite (called DaisySuite, see also Fig 2). DaisyGPS yields a configurable number of acceptors, donors and acceptor-like donors (default: 2, 3, 2). For each possible pair of acceptor and donor, a Daisy call is inferred. Daisy then tries to identify HGT regions for each acceptor-donor pair and reports them as candidates if the regions pass the thresholds defined in [16] for mapping coverage, number of split-reads and number of read pairs between acceptor and donor. Both pipelines can still be run independently. To unburden installation, we provide a setup script and provide DaisySuite components as Conda [31] packages. The simulations are also integrated into the DaisySuite pipeline (see DaisySuite documentation for details).

### Experimental setup

#### Data sets

We tested the complete DaisySuite on three types of data sets to validate both DaisyGPS and the integration with Daisy. The first type comprises the *H.pylori* data set, the KO11FL data set and the EHEC data set. All three were used in the Daisy publication (see [16] for detailed data set description) and are chosen as suitable ground truth and for the purpose of showing reproducibility. The second type comprises large-scale simulations analogous to the *H.pylori* simulation. Both positive (simulated HGT) and negative (no HGT) simulations are used to estimate sensitivity and specificity of the DaisySuite. In addition, varying evolutionary time frames and metagenomic samples contexts are simulated. In a third part, we use real data from an outbreak data set with 14 MRSA samples to elucidate further applicability of both DaisySuite. The details of the data sets and *in silico* experiments are explained below.

**H. pylori.** The data set *Helicobacter pylori* presents a simulated data set for a proof of principle already used for validation in the Daisy paper (see [16] for details of genomic simulation). The acceptor is *Escherichia coli* K12 substr. DH10B (NC_010473.1), the donor is *H. pylori* strain M1 (NZ_AP014710.1). The *in silico* transferred phage region of the *H. pylori* comprises a 28 Kbp region at the genomic positions 1 322 000—1 350 000. The insertion site within the acceptor is located at position 1 120 261.

**EHEC.** The HGT organism in the EHEC data set is *E.coli* O157:H7 Sakai [32] that derived from *E.coli* O55:H7 and is assumed to have acquired the Shiga-Toxins (Stx) via transduction from *Shigella dysenteriae*. According to literature, the bacteriophage carrying Stx is supposedly positioned at 2 643 556—2 694 691 in *E.coli* O55:H7. In [16] we proposed an alternative phage insertion site at 1 741 535—1 744 926.

**KO11FL.** The KO11FL data set comprises the transgenic *E.coli* KO11FL [33]. The acceptor is *E.coli* W, and the two donors are *Zymomonas mobilis* and the cloning vector pBEN77.

**Large-scale simulation.** We designed a large-scale simulation analogous to the *H.pylori* data set with positive and negative simulations. For each positive simulation, first an acceptor and a donor organism are randomly chosen among the available RefSeq sequences (date of retrieval: March 21, 2017, plasmids are ignored for sake of size consistency). A random 28 Kbp region is selected from the donor and inserted at a random position in the acceptor. The size 28 Kbp is chosen to systematically repeat the single simulation from the *H. pylori* example. Single nucleotide polymorphisms (SNPs) and small insertions and deletions (indels) are introduced separately into acceptor and donor region (SNP rate: 0.01, indel rate: 0.001). For each negative simulation, only an acceptor is randomly chosen, and SNPs and indels are introduced with the same rates as above. 150 bp reads are simulated from 500 bp fragments with 50 bp standard deviation with the Mason simulator [34]. The positive and negative simulations are repeated automatically 100 times.

**Simulations with varying mutation rate.** To assess the robustness of DaisySuite when handling more historic HGT events, we perform simulations on the *H. pylori* data set with growing mutation rates. Starting with a SNP rate of 0.01 and indel rate of 0.001, we increment both rates for 10 steps by 0.01 (SNPs) and 0.001 (indels), resulting in a maximal SNP rate of 0.1 and maximal indel rate of 0.01. Hence, we created 10 simulations by introducing both SNPs and small indels in corresponding rates, e.g. 0.01 SNPs and 0.001 small indels for the first sample, 0.02 SNPs and 0.002 small indels for the second sample, and so on. Each simulation step is repeated twice to avoid random artefacts.

**Simulations with metagenomic samples.** To show the applicability for metagenomic samples, we use data from the first CAMI (Critical Assessment of Metagenome Interpretation) challenge ([25], http://www.cami-challenge.org) to create three simulations with varying complexity regarding the number of organisms in a sample. The CAMI challenge provided three types of simulated data sets with varying complexity (*low*, *medium*, and *high*), i.e. the number of organisms per sample increases (40 to several hundred) with growing complexity. We choose one sample per complexity level (low: RL_S001__insert_270, medium: RM1_S001__insert_5000, high: RH_S001__insert_270). In all three cases, we spike in reads from the *H. pylori* data set. Both our data set and the CAMI challenge data sets are created from simulated Illumina 150 bp reads. For each data set from the CAMI challenge, we use 10% randomly sub-sampled reads ([35] showed no loss in sensitivity when profiling for this sub-sampling rate). We spike in 10% randomly sub-sampled reads from the *H. pylori* data set, resulting in an average 10x coverage for which the HGT site should still be detectable.

**MRSA outbreak.** The MRSA data set consists of 14 samples of methicillin resistant *Staphylococcus aureus* strains obtained during a MRSA outbreak at a neonatal intensive care unit (ENA accession number ERP001256, [36]). Seven samples are associated with the outbreak, labeled O1-O7 in this manuscript, the other seven samples N1-N7 are not associated with the outbreak. Sample description and run accession numbers are stated in the results section. Phylogenetic analysis by [36] separated the 14 samples into distinct groups according to their outbreak association. The reference isolate used in that study is the epidemic MRSA EMRSA-15 representative HO 5096 0412, and we use this as ground truth for acceptor candidates reported by DaisyGPS. The seven outbreak related MRSA samples have a distinct antimicrobial resistance pattern, and it is believed that the related resistance genes have been introduced via HGT. With DaisySuite we want to investigate if the outbreak strains share the same HGT regions and if they can be distinguished from the non-outbreak strains.

### Structure of validation

The setup of the validation is according to the types of data sets. In a first phase, we want to show a proof of concept given data with sufficient ground truth. The aim is to predict the correct acceptor and donor candidates with DaisyGPS and at the same time to reproduce the results obtained from Daisy. We therefore use the data sets already shown in the Daisy paper for sake of consistency. We set DaisyGPS to report a total of two acceptor candidates, four donor candidates, and two acceptor-like donor candidates for every data set and we evaluate if the correct acceptor and donor candidates are among them. For incorrect candidates of acceptor and donor, Daisy should not report HGT candidates unless the transferred region is present in multiple strains or there are multiple possible acceptors present with high sequence similarities as, e.g., among *E.coli* strains. For the EHEC data set, we activate the species filter since we are interested in strain candidates, and further blacklist taxa from the HGT organism to be analysed (*E.coli* O157:H7, taxon 83334) and the complete O157 lineage (parent taxon 1045010). For the KOFL11 data set, the HGT organism is blacklisted as well (*E.coli* KOFL11, taxon 595495). In a second part, we want to estimate the rate of sensitivity and specificity of the DaisySuite. We designed a large-scale simulation analogous to the *H.pylori* data set with positive and negative simulations (100 simulations each). From the positive simulations, we calculate the sensitivity for both DaisyGPS and Daisy (see below for definitions on metrics). DaisyGPS is designed with high sensitivity in mind and always reports the closest fitting candidates given sequencing data, even for non-HGT organisms. Hence, also for the negative simulations, DaisyGPS will report candidates and we expect a low specificity here. Daisy, however, should then report only few—if any—HGT candidates from the acceptor-donor pairs. Furthermore, we want to inspect how much the HGT-organism can mutate before the true acceptor and donor cannot be detected anymore. We use the *H. pylori* data set and insert SNPs and small indels at varying rates. We repeat this procedure two times for ten different mutation rates, resulting in a total of 20 data sets. We then check for each sample if DaisyGPS is still able to detect true acceptor and donor and if so, whether Daisy is able to detect the true HGT region. In addition, we want to estimate the applicability for metagenomic samples by using three simulated metagenomic samples with varying complexity that include reads from the *H. pylori* data set. DaisySuite should still report the correct acceptor and donor candidates for the *H. pylori* data set. MicrobeGPS is a metagenomic profiling tool and will hence report all organisms in the sample alongside the true acceptor and donor candidates. Hence, we have to adjust our settings and procedure for this analysis: To report more distinct candidates for downstream analysis, we increase the number of reported acceptor and donor candidates to 30, respectively, but set the maximal number of candidates per species to one. We only perform a follow up Daisy analysis for the true acceptor and donor—if the pair is reported. For metagenomic samples, we would generally recommend this procedure of separated DaisyGPS and Daisy runs while adjusting and trying different filter settings for DaisyGPS, and then only run Daisy on the most likely candidates.

In the last evaluation part, we test the DaisySuite on real data with unknown or uncertain ground truth. The MRSA outbreak data set consists of 14 samples, seven outbreak related and seven unrelated. Here we want to test if DaisySuite is able to distinguish between the outbreak and non-outbreak samples according to their reported acceptor, donor and HGT region candidates.

### Definition of evaluation metrics

The interpretation of various statistics depends on the hypothesis to be tested. In our analysis in the large-scale simulations, we differentiate between two scenarios: in the first one, we expect to detect an HGT event (positive test), while in the other one we assume the absence of an HGT event (negative test). For each simulation or run, a DaisyGPS call will lead to multiple pairs to be evaluated by Daisy. We therefore distinguish between statistics on runs and statistics on pairs that we will explain in the following.

For DaisyGPS, we consider during a positive test a single run as a true positive (TP) if the correct acceptor/donor pair is reported. Accordingly, a false negative (FN) occurs when the correct pair is not reported. Since the number of reported pairs is set by our settings, we will almost always have a fixed number of downstream verifications (except if there are not enough candidates to report) and thus we report the number of runs instead of pairs. Consequently, we can define the sensitivity as TP / #Runs. In a negative test setting, we deem those runs as true negatives (TNs) where either no pairs are reported or acceptor and donor of the pair are the very same organism. Note that if no other suitable candidates are available, the same organism may be reported as both acceptor and donor due to sorting by the respective scores, e.g. even an organism already reported as acceptor with a *donor*-*score* > 0 can be reported as donor if there is no candidate with a lower *donor*-*score*. All other pairs are regarded as FP that will each trigger an unnecessary verification in the downstream tools. Since we are interested in how many runs did not cause verifications, we can characterize the specificity by TN / #Runs. While it is obvious in both settings to rely on an exact match of the reported results and the ground truth, a reported organism still may be very close to the ground truth organism in terms of sequence similarity (negative and positive settings) and even include the very regions involved in the HGT event (positive setting). To account for this, we also use BLASTN in the case that no TP was reported and compare the FP to the ground truth. If the Blast identity of the FP to the ground truth is above 80% we change the classification from FP to BLAST-supported TP (Blast TP) since Daisy might still be able to infer the correct HGT region from these Blast TPs given the sufficient sequence similarity.

In Daisy, we evaluate acceptor/donor pairs and therefore the statistics are defined based on the condition of a pair reported by DaisyGPS. In a positive simulation, Daisy TP pairs are those that represent the correct pair and are detected by Daisy. It directly follows that each correct pair that is not supported by Daisy can be seen as a false negative (FN). Given that the pair is incorrect, i.e. a FP from DaisyGPS where the acceptor or donor is wrong, we count a rightly not supported pair as true negative (TN) and an erroneously detected pair as FP. To measure how many pairs are correctly identified, we define the sensitivity as (TP + TN) / #Pairs. Considering a negative test setting, we are mainly interested in the pairs that are wrongly reported as being involved in an HGT event. We declare those pairs as FP and describe the specificity as (#Pairs—FP) / #Pairs. It also follows that all the pairs that are not detected are TN. For a comprehensive summary of the classifications, refer to S1 Table.

Lastly, in the context of the complete DaisySuite pipeline, we evaluate the combined results of DaisyGPS and Daisy. Each pair reported by DaisyGPS for a single simulation induces an evaluation by Daisy. Since the overall result of the pipeline should indicate whether a simulation contains an HGT event or not, the classification of a DaisySuite run depends exclusively on the consolidated results of each Daisy evaluation for a single simulation. In a positive test setting, we want to find exactly the one pair that represents the HGT event. From that follows that a complete DaisySuite run can be classified as TP if Daisy supports solely the correct pair, i.e. Daisy reports the TP and no FP. This also implies that DaisyGPS needs to detect the TP. Similarly, in a negative test setting, a TN occurs if Daisy reports no HGT candidates at all.

### Settings and pre-/post-processing

DaisySuite is run with default parameters as of version 1.2.1 unless stated otherwise. The option to limit the maximum amount of candidates reported per species was introduced in version 1.3.0. The new version, however, did not introduce any changes to the used software versions, default parameters or other algorithmic aspects of DaisySuite. The parameter to combine potentially overlapping HGT candidates within Daisy is set to 20 bp, hence, overlapping regions with start and end positions differing by more than 20 bp are reported as separate candidates. For the comparison of the number and content of HGT sequences, we clustered overlapping HGT candidates with the tool usearch9 (v9.1.13_i86linux32) with identity 1.0 [37].

For validation, we determine the true presence of an HGT region in the samples by mapping the sample reads to all suggested, clustered regions with Bowtie2 (version 2.2.4). For comparison, we take the mean coverage of every region and apply a sigmoidal function to map all mean coverages to the [0.5,1] space for displaying a meaningful heatmap. The application of a sigmoidal function and the heatmap is computed in R (Rscript version 3.3.3). The heatmap function in R uses a hierarchical clustering with complete linkage as default, and we turned of the dendrogram for the columns. In addition, we perform a whole-genome alignment using the Mauve plugin (version 2.3.1) as part of the Geneious software (version 10.0.5) to establish shared HGT regions among the samples. To do this, we concatenate all HGT regions of a sample and separate the regions with segments of 1000*’N’ to avoid fragmented regions or overlapping local collinear blocks (LCBs).

## Results

### Acceptor and donor identification with DaisyGPS

In the first part of the validation, we test DaisyGPS on three data sets from simulated and real data with sufficient ground truth and already previously evaluated with Daisy. Since DaisySuite combines both tools, DaisyGPS and Daisy, the aim is to reproduce our previous results even without donor and acceptor being prespecified.

The *H.pylori* data set was simulated from *E.coli* K12 substr. DH10B as acceptor and *H. pylori* strain M1 as donor. DaisyGPS successfully reports both as such (see S2 and S3 Tables for complete candidate and HGT reports), and the subsequent Daisy run also reports the true HGT site. In addition to the only true HGT candidate previously already reported in the Daisy paper, DaisySuite reports another, FP HGT site for a region from *Haemophilus ducreyi*. The HGT region reported for *H. ducreyi* strain GHA9 has no continuous similarity with the HGT region from *H.pylori* (no blast hits longer than 15 bp, see S4 Table). However, the region on *H. ducreyi* shares the first 1200 bp and the last 1300 bp with the acceptor *E.coli* K12 substr. DH10B on multiple sites, and since beginning and end of the region are covered, almost six times as many split-reads are found as for the true acceptor site. The total coverage of the region is relatively low with 30x compared to 95x of the *H.pylori* but obviously high enough to pass the coverage filter.

The EHEC *E.coli* O157:H7 Sakai is supposedly derived by an HGT event where a defective prophage has been transferred from *Shigella dysenteriae* to *E.coli* O55:H7. Both are reported by DaisyGPS as candidates (see S5 Table). In line with its strong sequence similarity to the *E.coli* species, *S.dysenteriae* is labeled as an acceptor-like donor candidate. The proposed alternative HGT insertion site from our previous Daisy paper is still reported (see S6 Table).

The KO11FL data set comprises a transgenic *E.coli* W variant with transferred genes from *Zymomonas mobilis* and a plasmid that was not analysed here. DaisyGPS successfully reports *E.coli* W and *Zymomonas mobilis* as acceptor and donor candidates (see S7 Table). Daisy does not report any FP HGT candidates.

### Estimating sensitivity, specificity and robustness of DaisySuite through large-scale simulations

After validating DaisyGPS on data previously evaluated with Daisy as a proof of principle, we analyse DaisySuite in terms of robustness and sensitivity by performing a large-scale simulation. We perform the simulation for the *H.pylori* data set in a randomised and automated fashion generating 100 simulations with a transferred HGT region. To evaluate robustness, we also perform 100 negative simulations where an acceptor genome is simulated but no HGT region is inserted. With the positive simulations, we can estimate the sensitivity of the complete DaisySuite. For DaisyGPS, we evaluate how many from the 100 simulations have the correct acceptor and donor genome identified. Since DaisyGPS reports more than one potential acceptor-donor pair, we count a TP hit if the true pair is among them, and only count a FN if the true pair was not reported at all. In case the correct pair is not reported (acceptor or donor or both), we consider pairs with Blast sequence identity > 80% also as a potential HGT candidate pair, and also count them as a TP. To evaluate Daisy, we consider all pairs proposed by DaisyGPS.

For a true pair reported by DaisyGPS, Daisy can either report a TP HGT region or a FN if the region could not be identified. For an acceptor-donor pair wrongly proposed by DaisyGPS, Daisy can either report no HGT candidate region (TN) or a FP hit. When we summarise the DaisySuite results over all pairs of one simulation, we only count a TP for that simulation if Daisy did not report any FPs (despite any TPs or TNs).

Table 1 states the resulting counts for DaisyGPS and for the complete DaisySuite summarised over the 100 simulations. DaisyGPS yields a sensitivity of 79%. From the 79 TPs, 22 are based on either a wrong acceptor, or donor, or both but have still sufficient Blast similarity to the original acceptor or donor to be counted as TP according to our scoring. 69% of the TPs and FPs resulted in a TP or TN call from Daisy. It is noticeable that all DaisySuite FPs are Blast FPs.

**Table 1:** Positive HGT simulation. DaisyGPS calls correct acceptor and donor candidates with a sensitivity of 79%. The total sensitivity for DaisySuite from 100 HGT simulations regarding correct acceptor and donor candidates with a follow up correct HGT site call is 69%.

DaisyGPS				DaisySuite						
TP	Blast TP	FP	sensitivity	TP	Blast TP	TN	FP	Blast FP	FN	sensitivity
79	22	21	0.79	55	13	14	27	27	4	0.69

Table 2 states the number of reported pairs proposed by DaisyGPS and a detailed count based on each pair for Daisy. From the resulting 818 pairs, Daisy then reports the correct HGT region, or correctly no HGT region from a DaisyGPS FP, with a sensitivity of 89%.

**Table 2:** Positive HGT simulation. Daisy evaluates 818 pairs reported by DaisyGPS and calls the correct HGT region or correctly no HGT region with a sensitivity of 89%.

DaisyGPS	DaisySuite							
reported pairs	TP	Blast TP	TN	FP	Blast FP	FN	Blast FN	sensitivity
818	74	22	656	32	32	56	51	0.89

In addition to the positive simulations, we performed another 100 negative simulations where we randomly selected and variated an acceptor genome but did not insert any foreign region from a donor. DaisyGPS can now either produce a TN hit, i.e. report no candidates at all, or FP candidates. Since DaisyGPS is very sensitive by design, we expect it to generally report candidates and, hence, we want to estimate if these negative HGTs trigger reports by a Daisy follow-up call. As expected, the specificity for DaisyGPS is very low with 6% (see Table 3). However, Daisy reports only six FPs out of 743 pairs, i.e. three simulations produced a FP HGT report.

**Table 3:** Negative HGT simulation. For the 100 negative simulations, DaisyGPS correctly reports no acceptor and donor candidates for six simulations. From the 94 simulations causing a downstream evaluation with Daisy, only three lead to a FP call considering all outcomes from DaisySuite (summarised over the 100 simulations). Daisy evaluates 743 pairs and only has six FP HGT region calls in total over all those pairs.

DaisyGPS		DaisySuite		Daisy		
TN	specificity	FP	specificity	DaisyGPS pairs	FP	specificity
6	0.06	3	0.97	743	6	0.99

From these results we can infer that DaisySuite is able to distinguish HGT from non-HGT organisms and is very robust if no HGT is present.

### Evaluation of genetic divergence

To determine how robust our method is if the true acceptors and donors divert from the representative genome in the database, we performed a simulation over evolutionary distances by introducing increasing SNP and small indel rates into the *H. pylori* data set. We used the *H. pylori* data set to generate 20 simulations with varying mutation rates. We introduced both SNPs and indels starting with a rate of 0.01 and 0.001, respectively. We then incremented the rates by 0.01 (SNPs) and 0.001 (indels) for a total of 10 steps, yielding a maximum SNP rate of 0.1 and a maximum indel rate of 0.01. Each step was repeated twice to account for the randomness of mutations and read simulation.

Table 4 shows the results for the candidate detection by DaisyGPS. For this experiment, we used default settings, in particular, we report up to two acceptors and three donors. For up to 0.03 SNP rate and 0.003 indel rate, we can reliably determine the correct acceptor and donor as the top ranked candidates on strain level. Higher mutation rates obscur true acceptor by making other representatives of the *Enterobacteriaceae* family more similar to the HGT-organism, such that the true acceptor (on strain level) is not within the two highest ranking candidates anymore. For SNP rates 0.03-0.04 and indel rates 0.003-0.004, family representatives for *Enterobacteriaceae* are reported. For higher mutation rates, species representatives for *E. coli* are reported. For the ranks of the true acceptors and donors, please see S8 Table.

**Table 4:** Candidates for varying mutation rates. Each line indicates at which taxonomic level—if at all—the true acceptor and donor were reported among the top two candidates for a given SNP and small indel rate. * signals that in only one out of two repetitions the correct strain was reported.

SNP rate	Indel rate	TP Acceptor reported	TP Donor reported
0.01	0.001	strain	strain
0.02	0.002	strain	strain*
0.03	0.003	strain	strain
0.04	0.004	family	strain
0.05	0.005	family	strain*
0.06	0.006	species	strain
0.07	0.007	species	strain
0.08	0.008	species	strain
0.09	0.009	species	strain
0.1	0.01	species	None

In general, the donor can be detected on strain level even for higher rates. For SNP rates ranging from 0.01 to 0.09, we detect the true donor at least once among the three best candidates within two repetitions. This may be attributed to the fact that only a small part of the HGT organism stems from the donor and hence is less heavily altered by randomly distributed mutation events. For a SNP rate of 0.1, solely representatives of the species *E. coli* are reported, hence the true donor is not detectable.

To further investigate whether the reported candidates lead to an HGT region detection, we continued to run Daisy. For all data sets for which the true positive acceptor and donor were reported at strain level, Daisy could identify the correct location of the HGT event. Other *E. coli* strains likewise passed the thresholds and subsequently were also reported, although the true site was always the—or among the—highest scoring locations. The number of reported HGT sites increases the higher the mutation rates grow, and starting at a mutation rate of 0.04 (SNP) and 0.004 (indel), it can also be observed that the number of reported locations increases tremendously, making a practical evaluation infeasible. This clearly shows the limitations of the mapping-based approach with regards to genetic divergence, especially in such a highly represented and highly similar species as *E. coli*.

### Applicability for metagenomic samples

To evaluate the applicability for metagenomic samples, we use three simulated metagenomic data sets with spiked in reads from the *H.pylori* data set. The metagenomic data sets are from the CAMI challenge and have a varying complexity in terms of the number of contained organisms, classified as *low*, *medium*, and *high*. To account for the metagenomic context, we set the number of reported acceptors and donors to 30, respectively, and only report one candidate per species. The true *E.coli* K12 acceptor is among the top 20 ranked candidates (low rank 7, medium rank 8, high rank 18, see S9–S11 Tables for full lists of reported candidates), so a maximal number of 20 acceptor candidates would have been sufficient for identification even for the high complexity sample. Donor identification is more challenging due to the less amount of reads that can be assigned. Still, the true *H.pylori* donor is among the top 30 ranked candidates (low rank 12, medium rank 7, high rank 24). A follow-up Daisy run on the true acceptor-donor pair successfully reports the correct HGT region for all three complexities.

### Exploration of HGT detection with DaisySuite from MRSA outbreak data

MRSA strains are generally assumed to undergo HGT events frequently [38, 39]. The MRSA data set considered here consists of 14 samples with seven of them related to an MRSA outbreak (O1-O7) and seven MRSA samples not associated with the outbreak (N1-N7) but that occurred in the same time frame [36]. [36] analysed all 14 samples and compared them to the EMRSA-15 representative HO 5096 0412 as the supposedly closest relative of the outbreak strains. We first evaluate acceptor and donor candidates reported by DaisyGPS in relation to the proposed HO 5096 0412 reference and then investigate HGT region candidates reported by Daisy regarding a possible distinction of outbreak vs. non-outbreak samples. We activate the species filter as we are again interested in strain level candidates.

For all outbreak samples O1-O7, *S.aureus* HO 5096 0412 was reported as acceptor candidate by DaisyGPS (see Table 5 and S12–S39 Tables for individual results for each of the 14 MRSA data sets analysed). The same acceptor was also reported for non-outbreak samples N2, N6 and N7. Acceptor candidates for sample N1 are *S.aureus* ECT-R-2 and N315, for N3 and N4 *S.aureus* MSSA476 and MW2, and for N5 *S.aureus* MRSA252. Although not associated with the outbreak, samples N3 and N4 are from patients that shared the same room in the hospital where the outbreak occurred and hence are possibly related [36].

**Table 5:** Acceptor and number of HGT region candidates. For 10 of the 14 samples, EMRSA-15 (HO 5096 0412) was reported as acceptor candidate. This includes all outbreak samples. Column *HGT regions* states the number of reported HGT regions, and column *EMRSA-15 as acceptor for HGT regions* the respective number that were reported with HO 5096 0412 as acceptor.

Label	Isolate	Accession	EMRSA-15 as acceptor	HGT regions	EMRSA-15 as acceptor for HGT regions
O1	1B	ERR103401	x	4	4
O2	6C	ERR103403	x	4	3
O3	7C	ERR103404	x	5	3
O4	8C	ERR103405	x	3	3
O5	10C	ERR101899	x	4	4
O6	11C	ERR101900	x	1	1
O7	12C	ERR103394	x	5	3
N1	14C	ERR103395	-	5	-
N2	15C	ERR103396	x	2	2
N3	16B	ERR103397	-	4	-
N4	17B	ERR103398	-	4	-
N5	18B	ERR159680	-	5	-
N6	19B	ERR103400	x	7	5
N7	20B	ERR103402	x	2	2

The reported donors are largely the same for both outbreak and non-outbreak samples (see Table 6). No donor was reported exclusively for the outbreak samples but three donors only for non-outbreak strains N1, N4 and N6. These are *S.epidermidis* strains ATCC 12228 and PM221 as well as *Enterococcus faecium* Aus0004. Although *S.aureus* HO 5096 0412 was reported for all outbreak samples, there is no clear distinction in acceptor and donor candidates reported by DaisyGPS apart from the non-outbreak only donors.

**Table 6:** Reported donors summarised for all samples. Both outbreak associated and unassociated samples mostly report the same donor candidates with only few variations (see S12–S39 Tables for details). The only unique donors are reported for the unassociated samples N1, N4 and N6.

	Reported donors
Outbreak and non-outbreak	*S.pseudointermedius* ED99 and HKU10-03 *S.warneri* SG1 *S.epidermidis* RP62A *S.haemolyticus* JCSC1435 *S.aureus* COL *S.lugdunensis* HKU09-01
Non-outbreak only	*S.epidermidis* ATCC 12228 (N1,N6 only) and PM221 (N4 only) *E.faecium* Aus0004 (N1 only)

Table 5 states the total number of clustered HGT regions and the number of the clustered regions where HO 5096 0412 is the acceptor that are found by DaisySuite. Most HGT regions hence have the EMRSA-15 representative as acceptor.

**Fig 3:**
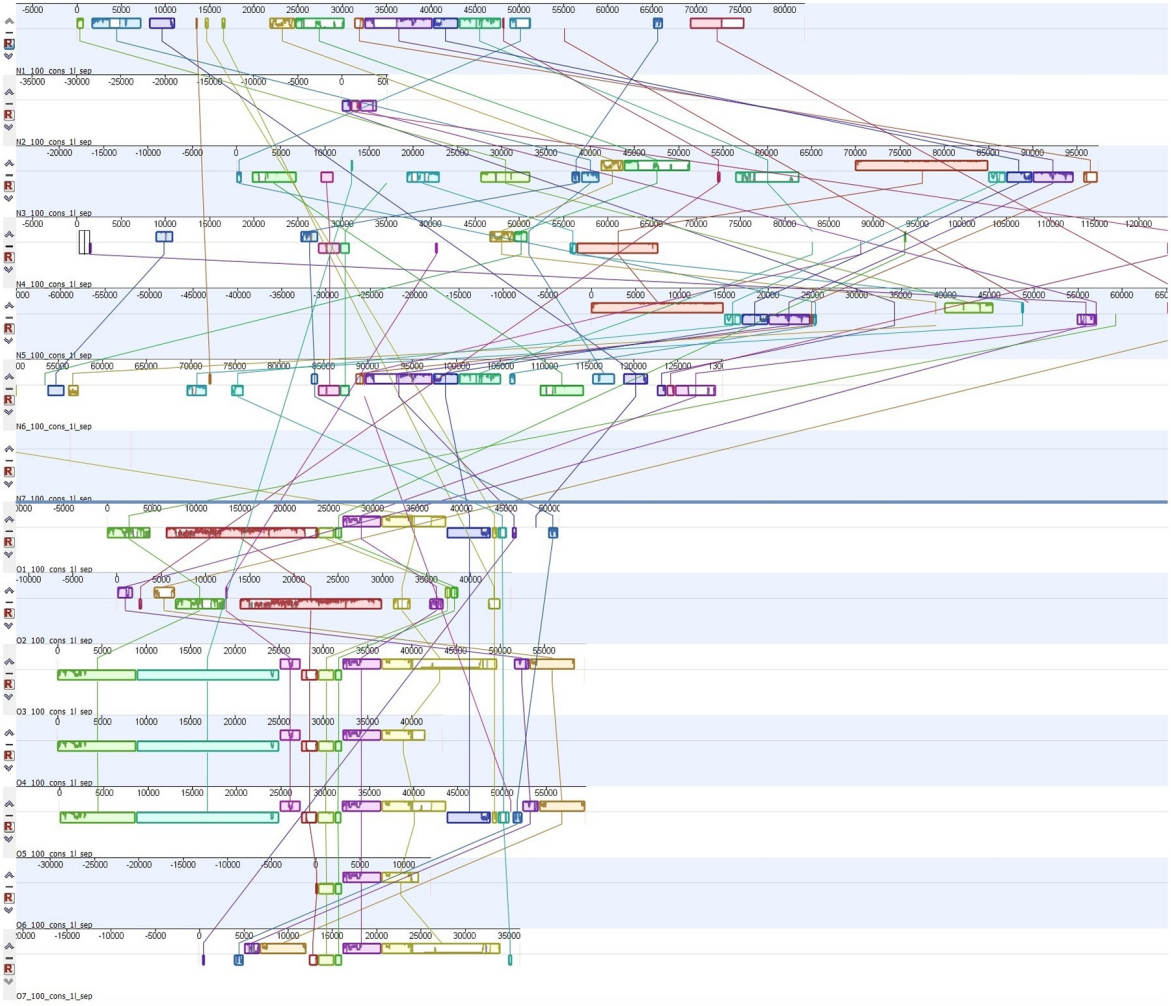
Mauve alignment of concatenated HGT regions. The HGT regions of all samples are aligned with Mauve to establish shared regions between them. The outbreak associated samples (O1-O7) in the lower part share most of their regions whereas the unassociated samples (N1-N7) in the upper part do not.

Fig 3 shows a Mauve alignment of the concatenated HGT regions of all 14 samples. There is a clear connection between the HGT regions from the lower seven samples O1-O7 that are the outbreak related samples. Samples N1-N7 also share some regions but do not have a clear connection as among the outbreak related strains. The overlap between outbreak and non-outbreak HGT regions is also low.

**Fig 4:**
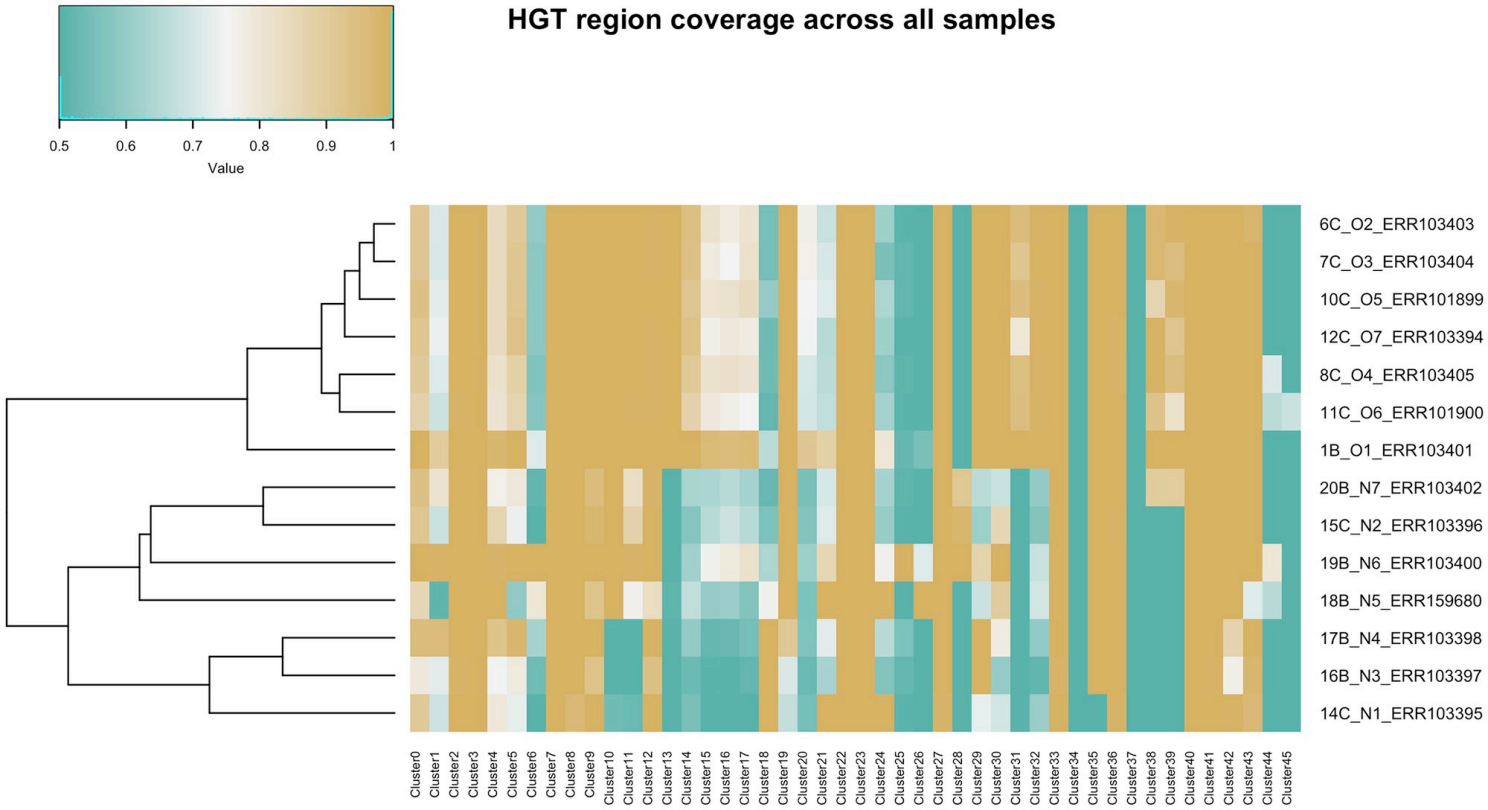
Heatmap of HGT region coverages. The mean coverages of HGT regions from all samples are calculated across every sample, and compared after application of a sigmoidal function. The order of the rows is obtained by the hierarchical clustering with complete linkage implemented in the sigmoidal function. Solid green spots indicate no coverage, solid ochre high coverage. Regions 34 and 37 are not covered in any sample and hence FP calls. Sample O6 shows presence of multiple HGT regions called by DaisySuite for other samples but missed here. There is a distinct presence of HGT regions between the outbreak samples in the upper part and the unassociated samples in the lower part.

Fig 4 shows the presence of the 41 HGT regions determined by mapping coverage called by Daisy among all samples. The purpose of the coverage analysis is to evaluate again if the HGT regions differ between the outbreak and non-outbreak strains but also to estimate if there are regions shared by all outbreak strains that are FN candidates of Daisy, or regions not covered at all that are likely FP candidates.

The clustering of samples according to the dendrogram shown in Fig 4 was done automatically (see settings part), and hence reflects the relation of the samples according to the mapping coverage of the proposed HGT regions.

All outbreak strains are clustered together and share most of their HGT regions. All non-outbreak strains for which DaisyGPS did not report EMRSA-15 as an acceptor candidate are clustered away furthest from the outbreak strains (N1, N3—N5). The likely related samples N3 and N4 are clustered together. Regarding a distinction of outbreak and non-outbreak strains, DaisySuite is able to determine the outbreak-related HGT regions which differ from the HGT candidates for the non-outbreak strains. Hence, a distinction is possible. Although DaisySuite only called one HGT region for O6, we can deduce from the coverage profile that more HGT regions called for the other outbreak samples are present as well but were missed by DaisySuite. As can be seen in the heatmap, clusters 34 and 37 are not covered by any sample and hence likely FPs. We detected the AMR gene *mecA* on Cluster 0, however, resistance is shared among all 14 samples according to [36]. No further AMR genes tested by [36] are detected on the other clusters. However, most of these AMR genes are on plasmids that were not analysed here.

## Discussion

We presented DaisyGPS, a pipeline that utilises metagenomic profiling strategies to identify acceptor and donor candidates from NGS reads of a potential HGT organism. DaisyGPS, together with Daisy, is part of the comprehensive HGT detection suite DaisySuite. We successfully validated DaisyGPS on simulated and real data previously analysed in [16]. We further demonstrated robustness of the DaisySuite on a large-scale simulation with 100 negative HGT tests, showing that DaisySuite correctly reports no HGT events with a specificity of 97%. On a large-scale simulation with 100 positive HGT simulations, DaisySuite reports the correct HGT event with a total sensitivity of 69%. From the 818 pairs reported by DaisyGPS among the 100 simulations, Daisy called the TP and TN regions with a sensitivity of 89%. Lastly, we evaluated DaisySuite on an MRSA outbreak data set with seven outbreak associated samples and seven not associated with the outbreak but that occurred during the same time frame. Here we could show that DaisySuite successfully distinguishes between associated and not associated samples regarding their suggested HGT regions, i.e. the outbreak samples show a distinct number and content of reported HGT regions.

One has to acknowledge that all outbreak strains have a high sequence similarity to the EMRSA-15 strain, which is not necessarily the case for the non-outbreak strains. This is also reflected in the results from DaisyGPS where *S.aureus* HO 5096 0412 is the best acceptor candidate for all outbreak strains but not reported at all for some non-outbreak strains. It directly follows that a sequence comparison based analysis as done with DaisySuite will likely find different patterns for the outbreak and non-outbreak strains, and a difference in HGT region candidates might seem obvious. However, starting from having established such a difference, there is value in then analysing the shared HGT region candidates among the outbreak-related strains. For this proof of concept, we performed a relatively simple evaluation by performing a coverage analysis of all HGT regions across all samples and investigating the presence of AMR genes within the HGT regions. But a future thorough follow-up analysis of the origin and functionality provided by the potential HGT sites could benefit our understanding of the risk and pathogenicity of these outbreak strains.

The observed FP and FN candidates, however, also reveal weaknesses of the sequence comparison approach. DaisyGPS is designed with a focus on sensitivity and hence inevitably leads to FP acceptor and donor candidate pairs to be examined by Daisy. Since these FPs are still due to a sufficient degree of mapping coverage, spurious split-reads and spanning reads can cause downstream FP calls as observed for the simulated data set from *E.coli K12* DH10 and *H.pylori*. The reported HGT site from *H.ducreyi* has only similarities in the start and end part of the proposed region compared to the transferred *H.pylori* region though. Insertion sites can also lie within repeat regions which enhances the negative impact of ambiguous mappings. This emphasises that a critical evaluation of HGT predictions is always crucial. To help interpret the HGT predictions from DaisySuite, the reported acceptor and donor candidates are ranked according to their respective score, and only the HGT sites passing the user defined thresholds (listed in the complete TSV results file) are reported in the final VCF results. In the supplementary results tables, we stated the parameters used for filtering or adjusting to the requirements of the data set. We also provide a documentation on usage at https://daisysuite.readthedocs.io/en/latest/tutorial/example.html.

From the missing HGT region calls for sample O6 that could be inferred from the coverage analysis, we can deduce that DaisySuite does not detect all HGT regions due to insufficient evidence. A potential cause could be that DaisyGPS did not report the correct donor reference. Even if DaisyGPS could find an appropriate donor genome, it is still likely that the genome content differs between the region present in the donor and the region actually present in the HGT organism. An alternative, complementary approach to cope with this problem of a lack of a suitable donor candidate could be to facilitate local, insertion sequence assembly. By offering identified insertion sequences, we can still provide the content of a potential HGT sequence and thereby enable downstream analysis. This approach would also support the detection of novel HGT sequences not present in current reference databases, and therefore also the detection of, e.g., novel antimicrobial resistance genes. Popins [40] is a tool for population-based insertion calling developed for human sequencing data (see, e.g., [41]). Popins only locally assembles unmapped reads (same input as for Daisy) with Velvet guided by a reference, thereby minimising the risk of potential misassemblies. On top of the assembly, Popins first uses spanning pairs (see red read pairs in Fig 1) to place an insertion in the (acceptor) reference, and then performs a local split-read alignment around the potential breakpoint. If multiple samples are provided, Popins merges contigs across samples into supercontigs, assuming that the same insertion is present in multiple samples. Although different bacterial samples do not represent a population as given for human populations, outbreak related samples still resemble a population such that one could use Popins for this purpose and gain valuable information. However, local insertion assembly only gives evidence for an insertion compared to the chosen acceptor reference, that does not necessarily mean that the insertion resulted from an HGT event. Hence, means to sophistically include insertion assembly results into the HGT context need to be defined first. Despite the evidence for an HGT event that DaisySuite can provide, the results should always be tested for alternative causations such as gene loss.

### Limitations

Our metagenomic analyses show that DaisySuite is able to detect HGTs not only from pure samples. However, the automatic detection of HGT events with DaisySuite in metagenomic samples has limitations if the diversity within the sample gets more complex. DaisyGPS uses the metagenomic classification tool MicrobeGPS, and hence, identifies organisms in the sample as part of the pipeline. All identified organisms with a homogeneous coverage are—per se—possible acceptor candidates. We increased the thresholds for the reported acceptor and also donor candidates to 30 entries, respectively, and limited the number of candidates per species to one so that the ground truth acceptor and donor of the simulated *H. pylori* are still listed. Note that this number not only depends on the number of organisms in the sample but also on their sequence similarity—especially to the expected acceptor and donor candidates.

The resulting 400 Daisy runs would require too much compute time and space for a systematic and automatic follow up. In general for metagenomic samples, we would recommend to only run DaisyGPS first and then define a confined set of likely candidates for follow up analysis. For future developments, we would suggest to integrate another mapping-based filtering for this definition where we would search for likely pairs via paired-end reads with one read mapping to an acceptor and the other to a donor candidate. We use this criterion also in the Daisy follow up as evidence but in our opinion it would also serve well for candidate (pair) filtering.

[42] applied a method that is similar to Daisy to detect mobile genetic elements (MGEs) in the human gut microbiome. Although this study shows the general applicability of our approach in a large scale metagenomic study, the focus here can only be the collection of now present or absent MGEs in the microbiome (rather than particular strains). [42] also point out that such a MGE characterisation is more meaningful in a time series analysis rather than from a single sample snapshot. Daisy has also been applied to infer horizontally transferred genes in the Daphnia iridescent virus 1 [43] which shows that our approach can be further applied in other contexts than bacteria.

DaisySuite uses mapping-based similarity to determine candidates. This can lead to biases if the true candidates are missing in the database or for historic events that are obscured through amelioration. DaisyGPS will still report the next best candidates (i.e. with the most sequence similarity) but the FPs in our large scale simulation arising from Blast hits already show the potential for downstream errors. Further, our simulation over evolutionary distances clearly show the limitations for acceptor and donor identification above a certain distance. This limitation also goes hand in hand with a sufficient sequencing coverage to avoid further bias by random sequencing errors, and also to allow a reliable Daisy follow-up analysis. From our experiments, we would recommend to provide at least a 10x sequencing coverage.

DaisySuite facilitates the capabilities of programs designed for different tasks, including mapping, metagenomic profiling and structural variant detection. Although this allows us to combine the strength of each tool to tackle the problem of HGT detection, we are also vulnerable to bottlenecks regarding the runtime of single steps. In particular, data sets that create big mapping results and/or contain many split reads may increase the runtime significantly. In general, the overall runtime ranges around one to two hours on a standard machine to process a standard sample, e.g., the *H. pylori* data set. However, very big or diverse data sets, such as created in our genetic divergence experiment, will increase the runtime manifold and in extreme cases render them infeasible to run. The main bottleneck for DaisyGPS is the metagenomic profiling via MicrobeGPS, whereas for Daisy the split read detection by Gustaf and—if Gustaf detects enough split reads—the HGT detection itself. In the future, we hope to alleviate this problem by modernising or helping to modernise the respective tools.

As with all computational methods, they cannot fully replace critical human thinking and should be cross validated by other means. In an HGT detection study, we would recommend to use other HGT detection methods (computational and/or wet lab) to support findings by individual methods. Although we see this as crucial, we think it lies outside the scope of DaisySuite to provide such a cross validation.

### Conclusion

With DaisyGPS, we present a tool for acceptor and donor identification from NGS reads of an HGT organism. To do that, DaisyGPS refines metrics already defined and used for metagenomic profiling purposes to account for the acceptor and donor specific coverage profiles. We integrated DaisyGPS with Daisy into a comprehensive HGT detection suite, called DaisySuite, that provides an automatic workflow to first determine acceptor and donor candidates and then identify and characterise HGT regions from the suggested acceptor-donor pairs. We successfully evaluated DaisyGPS on data previously analysed with Daisy, and demonstrated sensitivity and robustness of the DaisySuite in a large-scale simulation with 100 simulated positive and negative HGT events. We could further show the benefits of an HGT analysis with DaisySuite on an MRSA outbreak data set where DaisySuite reported HGT candidates that help to distinguish between outbreak associated and unassociated samples and therefore also provide information for outbreak strain characterisation.

## Supporting information

S1 Table Confusion matrix for DaisyGPS and Daisy classifications. (PDF)

S2 Table Acceptor and donor candidates for the *H. pylori* data set. (PDF)

S3 Table Daisy results for the *H. pylori* data set. (PDF)

S4 Table Blast hits of *H. pylori* HGT region reported for *H.ducreyi* strain GHA9. (PDF)

S5 Table Acceptor and donor candidates for the EHEC data set. (PDF)

S6 Table Daisy results for the EHEC data set. (PDF)

S7 Table Acceptor and donor candidates for the KO11FL data set. (PDF)

S8 Table Ranks of true acceptor and donor in the *H. pylori* genetic evolution experiment. (PDF)

S9 Table Acceptor and donor candidates for the CAMI low complexity data set. (PDF)

S10 Table Acceptor and donor candidates for the CAMI medium complexity data set. (PDF)

S11 Table Acceptor and donor candidates for the CAMI high complexity data set. (PDF)

S12 Table Acceptor and donor candidates for MRSA data set (ERR103401). (PDF)

S13 Table Daisy results for MRSA data set (ERR103401). (PDF)

S14 Table Acceptor and donor candidates for MRSA data set (ERR103403). (PDF)

S15 Table Daisy results for MRSA data set (ERR103403). (PDF)

S16 Table Acceptor and donor candidates for MRSA data set (ERR103404). (PDF)

S17 Table Daisy results for MRSA data set (ERR103404). (PDF)

S18 Table Acceptor and donor candidates for MRSA data set (ERR103405). (PDF)

S19 Table Daisy results for MRSA data set (ERR103405). (PDF)

S20 Table Acceptor and donor candidates for MRSA data set (ERR101899). (PDF)

S21 Table Daisy results for MRSA data set (ERR101899). (PDF)

S22 Table Acceptor and donor candidates for MRSA data set (ERR101900). (PDF)

S23 Table Daisy results for MRSA data set (ERR101900). (PDF)

S24 Table Acceptor and donor candidates for MRSA data set (ERR103394). (PDF)

S25 Table Daisy results for MRSA data set (ERR103394). (PDF)

S26 Table Acceptor and donor candidates for MRSA data set (ERR103395). (PDF)

S27 Table Daisy results for MRSA data set (ERR103395). (PDF)

S28 Table Acceptor and donor candidates for MRSA data set (ERR103396). (PDF)

S29 Table Daisy results for MRSA data set (ERR103396). (PDF)

S30 Table Acceptor and donor candidates for MRSA data set (ERR103397). (PDF)

S31 Table Daisy results for MRSA data set (ERR103397). (PDF)

S32 Table Acceptor and donor candidates for MRSA data set (ERR103398). (PDF)

S33 Table Daisy results for MRSA data set (ERR103398). (PDF)

S34 Table Acceptor and donor candidates for MRSA data set (ERR159680). (PDF)

S35 Table Daisy results for MRSA data set (ERR159680). (PDF)

S36 Table Acceptor and donor candidates for MRSA data set (ERR103400). (PDF)

S37 Table Daisy results for MRSA data set (ERR103400). (PDF)

S38 Table Acceptor and donor candidates for MRSA data set (ERR103402). (PDF)

S39 Table Daisy results for MRSA data set (ERR103402). (PDF)
