# Supplementary material for: Where did you come from, where did you go: Refining metagenomic analysis tools for horizontal gene transfer characterisation
Source: PLoS Comput Biol. 2019 Jul 23;15(7):e1007208. doi: 10.1371/journal.pcbi.1007208 (PMC6677323; doi:10.1371/journal.pcbi.1007208)
Supplement: S1 Table — (PDF) [file pcbi.1007208.s001.pdf]

S1 Table: Confusion matrix for **a)** DaisyGPS and **b)** Daisy classifications. **a)** If the simulation contains an HGT and DaisyGPS reports at least one candidate pair that corresponds to the correct acceptor/donor pair, the run is considered a TP. If DaisyGPS fails to report the correct acceptor or donor, the run is deemed a FP since all pairs will undergo follow up analysis by Daisy. In a negative test setting, a FP occurs if DaisyGPS reports any pair where the acceptor does not equal the donor and a TN means that either no pair was reported or acceptor and donor of the pair are the same organism. **b)** If the pair represents a HGT and Daisy reports it, the pair is classified as TP. If it is not reported, the run is considered a FP. Analogously, if the pair is no HGT, but Daisy reports a HGT event taking place, the pair counts as FP, otherwise as TN.

a)

		True condition (ground truth)	
		Simulation contains HGT (positive setting)	Simulation does not contain HGT (negative setting)
Predicted condition (DaisyGPS)	Run reports HGT	TP	FP
	Run does not report any HGT	FP	TN

b)

		True condition (ground truth)	
		Pair represents HGT (DaisyGPS TP)	Pair does not represent HGT (DaisyGPS FP)
Predicted condition (Daisy)	Pair reports HGT	TP	FP
	Pair does not report any HGT	FP	TN
