# Supplementary material for: Where did you come from, where did you go: Refining metagenomic analysis tools for horizontal gene transfer characterisation
Source: PLoS Comput Biol. 2019 Jul 23;15(7):e1007208. doi: 10.1371/journal.pcbi.1007208 (PMC6677323; doi:10.1371/journal.pcbi.1007208)
Supplement: S2 Table — (PDF) [file pcbi.1007208.s002.pdf]

S2 Table: Results of the DaisyGPS run for the *H. pylori* data set with yara, no species filter and no samflag filter. Sampling sensitivity = 90. No taxon blacklist. No parent blacklist. No species blacklist. (-)0.000^{*} represents absolute values $< 0.000\bar{4}$. The true positive acceptor and donor are marked in bold. [1]Salmonella enterica subsp. enterica serovar Anatum str. USDA-ARS-USMARC-1676

Candidate			MicrobeGPS metrics			DaisyGPS metrics	
Type	Name	Accession.Version	Number Reads	Validity	Heterogeneity	Donor Score	Acceptor Score
**Acceptor**	**Escherichia coli str. K-12 substr. DH10B**	**NC_010473.1**	**197800**	**0.254**	**0.082**	**0.173**	**0.003**
Acceptor	Escherichia coli K-12	NZ_CP010445.1	187050	0.237	0.075	0.162	0.003
Donor	[Haemophilus] ducreyi	NZ_CP015434.1	322	0.001	0.926	-0.924	-0.000*
Donor	Salmonella enterica [...] USDA-ARS-USMARC-1676[1]	NZ_CP014620.1	126	0.001	0.919	-0.918	-0.000*
Donor	Klebsiella oxytoca KONIH1	NZ_CP008788.1	1791	0.001	0.795	-0.794	-0.000*
**Donor**	**Helicobacter pylori**	**NZ_AP014710.1**	**9154**	**0.018**	**0.79**	**-0.782**	**-0.001**
Acceptor-like Donor	Escherichia coli	NZ_CP016182.1	74580	0.094	0.088	0.006	0.000*
