# Supplementary material for: Where did you come from, where did you go: Refining metagenomic analysis tools for horizontal gene transfer characterisation
Source: PLoS Comput Biol. 2019 Jul 23;15(7):e1007208. doi: 10.1371/journal.pcbi.1007208 (PMC6677323; doi:10.1371/journal.pcbi.1007208)
Supplement: S3 Table — (PDF) [file pcbi.1007208.s003.pdf]

S3 Table: Results of the Daisy run for the *H. pylori* data set run with yara, gustaf, no species filter and no samflag filter. Sampling sensitivity = 90. Split read threshold = 3. No taxon blacklist. No parent blacklist. No species blacklist. The true positive HGT region is marked in bold.

Organism		Acceptor			Donor			Read Evidence			Evidence Filter			
Acceptor	Donor	Start	End	Coverage	Start	End	Coverage	Split	Spanning	Within	A-Cov	D-Cov	Spanning	Within
NZ_CP010445.1	NZ_AP014710.1	1880235	1880237	44.0	1322002	1350000	94.62	152	182	8712	7	100	100	100
NZ_CP010445.1	NZ_CP015434.1	3904873	3904886	40.54	114928	126957	30.41	871	156	884	3	100	100	100
**NC_010473.1**	**NZ_AP014710.1**	**1120261**	**1120263**	**43.0**	**1322002**	**1350000**	**94.62**	**154**	**182**	**8712**	**3**	**100**	**100**	**100**
