# Supplementary material for: Where did you come from, where did you go: Refining metagenomic analysis tools for horizontal gene transfer characterisation
Source: PLoS Comput Biol. 2019 Jul 23;15(7):e1007208. doi: 10.1371/journal.pcbi.1007208 (PMC6677323; doi:10.1371/journal.pcbi.1007208)
Supplement: S4 Table — (PDF) [file pcbi.1007208.s004.pdf]

S4 Table: Blastn hits for HGT region reported for *H.ducreyi* strain GHA9 (NZ_CP015434.1) against original HGT region from *H.pylori* (NZ_AP014710.1) and the original acceptor *E.coli* K12 substr. DH10B (NC_010473.1). Only Blast results for *E.coli* K12 substr. DH10B with at least 100 bp and 90% identity are shown.

query acc.ver	subject acc.ver	% identity	alignment length	mismatches	gap opens	q. start	q. end	s. start	s. end	evalue	bit score
NZ_CP015434.1	NZ_AP014710.1	100.000	15	0	0	9575	9589	7203	7217	0.99	28.3
NZ_CP015434.1	NZ_AP014710.1	100.000	14	0	0	7602	7615	10086	10099	3.5	26.5
NZ_CP015434.1	NZ_AP014710.1	100.000	14	0	0	2556	2569	12036	12049	3.5	26.5
NZ_CP015434.1	NZ_AP014710.1	100.000	14	0	0	2289	2302	22068	22081	3.5	26.5
NZ_CP015434.1	NZ_AP014710.1	100.000	14	0	0	4872	4885	22081	22068	3.5	26.5
NZ_CP015434.1	NC_010473.1	100.000	1330	0	0	10696	12025	3200797	3199468	0.0	2399
NZ_CP015434.1	NC_010473.1	99.249	1332	10	0	10693	12024	4640586	4641917	0.0	2358
NZ_CP015434.1	NC_010473.1	98.724	1332	17	0	10693	12024	3213260	3211929	0.0	2325
NZ_CP015434.1	NC_010473.1	99.917	1199	1	0	1	1199	3211931	3213129	0.0	2158
NZ_CP015434.1	NC_010473.1	99.917	1199	1	0	1	1199	4641915	4640717	0.0	2158
NZ_CP015434.1	NC_010473.1	99.249	1199	9	0	1	1199	3199471	3200669	0.0	2122
