# Supplementary material for: Where did you come from, where did you go: Refining metagenomic analysis tools for horizontal gene transfer characterisation
Source: PLoS Comput Biol. 2019 Jul 23;15(7):e1007208. doi: 10.1371/journal.pcbi.1007208 (PMC6677323; doi:10.1371/journal.pcbi.1007208)
Supplement: S5 Table — (PDF) [file pcbi.1007208.s005.pdf]

S5 Table: Results of the DaisyGPS run for the EHEC data set run with yara, species filter and no samflag filter. Taxon blacklist: [83334, 1045010]. Parent blacklist: [83334]. No species blacklist. (-)0.000^* represents absolute values $< 0.000\bar{4}$. The true positive acceptor and donor are marked in bold.

Candidate			MicrobeGPS metrics			DaisyGPS metrics	
Type	Name	Accession.Version	Number Reads	Validity	Heterogeneity	Donor Score	Acceptor Score
Acceptor	Escherichia coli Xuzhou21	NC_017906.1	1040394	0.846	0.054	0.792	0.018
**Acceptor**	**Escherichia coli O55:H7 str. RM12579**	**NC_017656.1**	**816492**	**0.723**	**0.040**	**0.683**	**0.012**
Donor	Cronobacter sakazakii CMCC 45402	NC_023032.1	201	0.006	0.861	-0.855	-0.000*
Donor	Enterobacter hormaechei subsp. hormaechei	NZ_CP010377.1	206	0.002	0.78	-0.778	-0.000*
Donor	Citrobacter freundii CFNIH1	NZ_CP007557.1	1443	0.001	0.743	-0.742	-0.000*
Donor	Citrobacter koseri ATCC BAA-895	NC_009792.1	93	0.004	0.560	-0.557	-0.000*
Acceptor-like Donor	Corynebacterium humireducens NBRC 106098 = DSM 45392	NZ_CP005286.1	117	0.444	0.078	0.366	0.000*
**Acceptor-like Donor**	**Shigella dysenteriae Sd197**	**NC_007606.1**	**148868**	**0.193**	**0.041**	**0.152**	**0.001**
