# Supplementary material for: Where did you come from, where did you go: Refining metagenomic analysis tools for horizontal gene transfer characterisation
Source: PLoS Comput Biol. 2019 Jul 23;15(7):e1007208. doi: 10.1371/journal.pcbi.1007208 (PMC6677323; doi:10.1371/journal.pcbi.1007208)
Supplement: S6 Table — (PDF) [file pcbi.1007208.s006.pdf]

S6 Table: Results for the EHEC data set run with yara, gustaf, species filter and no samflag filter. Sampling sensitivity = 95. Split read threshold = 3. Taxon blacklist: [83334, 1045010]. Parent blacklist: [83334]. No species blacklist. Results (139 HGT candidates) for NC_017656.1 (acceptor) and NZ_CP007557.1 (donor) are omitted here for sake of simplicity. For all other pairs no HGT candidates were reported.

Organism		Acceptor			Donor			Read Evidence			Evidence Filter			
Acceptor	Donor	Start	End	Coverage	Start	End	Coverage	Split	Spanning	Within	A-Cov	D-Cov	Spanning	Within
NC_017656.1	NC_007606.1	314439	334641	27.39	2213697	2214454	63.18	39	3	102	0	100	100	100
NC_017656.1	NC_007606.1	1570633	1580081	138.85	1282007	1320884	7.51	9	1	714	100	97	96	98
NC_017656.1	NC_007606.1	1570633	1584983	141.99	1282007	1329491	11.14	11	12	973	99	97	98	97
NC_017656.1	NC_007606.1	1580080	1584983	148.04	1320883	1329491	27.6	8	12	261	99	99	99	99
NC_017656.1	NC_007606.1	1589216	1618452	247.73	4032919	4035786	110.69	107	10	576	100	100	100	100
NC_017656.1	NC_007606.1	1738741	1739271	30.87	1321240	1322115	88.45	42	73	60	4	100	100	98
NC_017656.1	NC_007606.1	1738741	1739785	157.15	1321240	1322656	58.2	17	5	72	95	100	100	99
NC_017656.1	NC_007606.1	1738741	1740010	134.9	1321240	1322870	51.13	50	3	72	96	100	100	98
NC_017656.1	NC_007606.1	1738741	1740078	129.54	1321240	1322973	49.81	17	6	81	100	98	100	98
NC_017656.1	NC_007606.1	1738741	1745278	119.31	1321240	1331304	23.91	9	52	202	96	98	100	98
NC_017656.1	NC_007606.1	1739270	1739785	287.13	1322114	1322656	9.33	56	5	13	99	96	100	99
NC_017656.1	NC_007606.1	1739270	1740477	130.81	1322114	1323341	21.27	28	3	42	96	98	99	98
NC_017656.1	NC_007606.1	1739270	1745278	127.11	1322114	1331304	17.77	24	52	143	97	99	99	96
NC_017656.1	NC_007606.1	1739784	1741539	10.67	1283675	1322655	11.22	19	294	897	4	97	100	97
NC_017656.1	NC_007606.1	1739784	1745278	112.11	1322655	1331304	18.29	16	51	130	95	100	100	100
NC_017656.1	NC_007606.1	1740009	1740477	6.25	1322869	1323341	42.62	20	3	28	5	97	100	96
NC_017656.1	NC_007606.1	1740009	1745278	115.53	1322869	1331304	18.65	17	52	129	98	99	100	96
NC_017656.1	NC_007606.1	1740077	1740477	2.25	1322972	1323341	46.64	16	3	25	4	100	100	100
NC_017656.1	NC_007606.1	**1741538**	**1744925**	164.13	**1283674**	**1288080**	59.4	18	9	692	99	100	100	100
NC_017656.1	NC_007606.1	1741538	1745278	159.71	1283674	1331304	12.51	9	166	1031	100	97	99	95
NC_017656.1	NC_007606.1	1957909	1958879	132.94	4032919	4035786	110.69	41	7	576	99	99	98	99
NC_017656.1	NC_007606.1	1957909	1982375	118.01	4032919	4035782	110.56	17	12	576	97	100	100	100
NC_017656.1	NC_007606.1	1958870	1982375	117.37	4034933	4035782	356.29	22	35	576	98	100	100	100
NC_017656.1	NC_007606.1	1986050	1986053	726.33	1288361	1331322	7.47	10	335	319	100	97	100	95
NC_017656.1	NC_007606.1	1986050	1992463	155.63	1321775	1331322	25.25	126	72	197	99	98	100	97
NC_017656.1	NC_007606.1	1986234	1992463	146.03	1321775	1329808	28.06	261	80	190	100	98	100	96
NC_017656.1	NC_007606.1	1986234	1992955	155.68	1320887	1329808	32.55	35	126	308	99	100	100	99
NC_017656.1	NC_007606.1	1992462	1992955	277.57	1320887	1321774	73.17	131	91	106	100	99	100	99
NC_017656.1	NC_007606.1	2431977	2443616	15.53	1282008	1322832	10.76	17	60	897	0	96	100	96
NC_017656.1	NC_007606.1	2435781	2443492	8.8	1282069	1320883	7.51	193	62	714	3	98	98	98
NC_017656.1	NC_007606.1	2469232	2481815	49.5	4032919	4035785	110.66	81	24	576	2	99	100	99
NC_017656.1	NC_007606.1	2486033	2488461	149.98	4298967	4301718	16.95	23	5	67	95	97	100	96
NC_017656.1	NC_007606.1	2486033	2488662	150.6	4298967	4301905	16.19	65	10	68	99	98	100	98
NC_017656.1	NC_007606.1	2486203	2488662	153.24	4299043	4301905	16.62	47	10	68	99	97	100	95
NC_017656.1	NC_007606.1	2486203	2488723	152.86	4299043	4301977	17.39	29	10	69	98	96	100	95
NC_017656.1	NC_007606.1	2487505	2489413	150.49	953376	956244	23.61	10	3	119	98	98	99	99
NC_017656.1	NC_007606.1	2488461	2489413	130.37	953376	954653	52.39	12	4	119	95	99	100	97
NC_017656.1	NC_007606.1	2488601	2488723	136.13	4301842	4301977	32.39	8	11	2	98	97	100	96
NC_017656.1	NC_007606.1	2678766	2679015	44.61	1323123	1323370	29.6	18	4	5	5	97	100	96
NC_017656.1	NC_007606.1	3607310	3629241	31.35	4189699	4189800	491.86	42	24	12	0	100	100	98
NC_017656.1	NC_007606.1	3615738	3630353	153.33	4195901	4198011	700.99	149	6	4245	97	100	98	100
NC_017656.1	NC_007606.1	3615738	3632904	131.04	4195901	4206697	139.78	21	4	4245	96	100	98	100
NC_017656.1	NC_007606.1	3615738	3632993	130.65	4195901	4206818	138.38	19	4	4250	98	100	98	100
NC_017656.1	NC_007606.1	3629240	3630353	1409.38	4189698	4198011	184.22	222	38	4278	100	100	100	100
NC_017656.1	NC_007606.1	3629240	3632904	430.46	4189698	4206697	91.85	30	36	4278	100	100	100	100
NC_017656.1	NC_007606.1	3629240	3632993	421.57	4189698	4206818	91.3	27	36	4283	100	100	100	100
