# Supplementary material for: Where did you come from, where did you go: Refining metagenomic analysis tools for horizontal gene transfer characterisation
Source: PLoS Comput Biol. 2019 Jul 23;15(7):e1007208. doi: 10.1371/journal.pcbi.1007208 (PMC6677323; doi:10.1371/journal.pcbi.1007208)
Supplement: S7 Table — (PDF) [file pcbi.1007208.s007.pdf]

S7 Table: Acceptor and donor candidates for the KO11FL data set run with yara, no species filter and no samflag filter. Taxon blacklist: [595495]. No parent blacklist. No species blacklist. (-)0.000^* represents absolute values $< 0.000\bar{4}$. The true positive acceptor and donor are marked in bold.

Candidate			MicrobeGPS metrics			DaisyGPS metrics	
Type	Name	Accession.Version	Number Reads	Validity	Heterogeneity	Donor Score	Acceptor Score
Acceptor	Escherichia coli W	NC_017635.1	221389	0.852	0.024	0.829	0.026
**Acceptor**	**Escherichia coli W**	**NC_017664.1**	**221570**	**0.853**	**0.025**	**0.828**	**0.026**
Donor	Salmonella enterica subsp. enterica serovar Infantis	NZ_CP016410.1	83	0.005	0.943	-0.938	-0.000*
Donor	[Haemophilus] ducreyi	NZ_CP015434.1	119	0.001	0.920	-0.919	-0.000*
**Donor**	**Zymomonas mobilis subsp. mobilis NRRL B-12526**	**NZ_CP003709.1**	**3067**	**0.002**	**0.876**	**-0.874**	**-0.000***
Acceptor-like Donor	Shigella boydii CDC 3083-94	NC_010658.1	23506	0.150	0.047	0.104	0.000*
Acceptor-like Donor	Shigella sonnei 53G	NC_016822.1	29127	0.168	0.073	0.095	0.000*
