# Supplementary material for: Where did you come from, where did you go: Refining metagenomic analysis tools for horizontal gene transfer characterisation
Source: PLoS Comput Biol. 2019 Jul 23;15(7):e1007208. doi: 10.1371/journal.pcbi.1007208 (PMC6677323; doi:10.1371/journal.pcbi.1007208)
Supplement: S8 Table — (PDF) [file pcbi.1007208.s008.pdf]

S8 Table: Ranks of the true acceptor and donor in the *H. pylori* genetic evolution data set. Ranks for both repetitions are shown.

SNP Rate	Indel Rate	TP Acceptor Ranks		TP Donor Ranks	
0.01	0.001	1	1	1	2
0.02	0.002	1	1	2	4
0.03	0.003	1	1	1	2
0.04	0.004	216	125	2	2
0.05	0.005	337	335	3	49
0.06	0.006	342	326	1	1
0.07	0.007	240	231	1	1
0.08	0.008	185	218	1	1
0.09	0.009	119	49	1	1
0.1	0.01	49	50	NA	NA
