# Supplementary material for: Where did you come from, where did you go: Refining metagenomic analysis tools for horizontal gene transfer characterisation
Source: PLoS Comput Biol. 2019 Jul 23;15(7):e1007208. doi: 10.1371/journal.pcbi.1007208 (PMC6677323; doi:10.1371/journal.pcbi.1007208)
Supplement: S9 Table — (PDF) [file pcbi.1007208.s009.pdf]

S9 Table: Acceptor and donor candidates for cami low complexity 10% sub-sampling and *H. pylori* 10% sub-sampling run with yara, one candidate per species, no species filter and no samflag filter. Sampling sensitivity = 90. No taxon blacklist. No parent blacklist. No species blacklist. (-)0.000^* represents absolute values $< 0.000\bar{4}$. The true positive acceptor and donor of the spiked in HGT organism are marked in bold.

Candidate			MicrobeGPS metrics			DaisyGPS metrics	
Type	Name	Accession.Version	Number Reads	Validity	Heterogeneity	Donor Score	Acceptor Score
Acceptor	Pseudomonas aeruginosa DSM 50071	NZ_CP012001.1	411552	0.99	0.003	0.987	0.018
Acceptor	Paracoccus denitrificans PD1222	NC_008686.1	105227	0.983	0.032	0.951	0.005
Acceptor	Xylella fastidiosa subsp. fastidiosa GB514	NC_017562.1	9952	0.911	0.011	0.900	0.000*
Acceptor	Desulfatibacillum alkenivorans AK-01	NC_011768.1	26663	0.317	0.038	0.28	0.000*
Acceptor	Nonlabens dokdonensis DSW-6	NC_020156.1	4139	0.976	0.014	0.961	0.000*
Acceptor	Exiguobacterium sp. ZWU0009	NZ_CP018057.1	37171	0.182	0.096	0.086	0.000*
**Acceptor**	**Escherichia coli str. K-12 substr. DH10B**	**NC_010473.1**	**19729**	**0.215**	**0.08**	**0.136**	0.000*
Acceptor	Exiguobacterium sp. U13-1	NZ_CP015731.1	31829	0.168	0.098	0.070	0.000*
Acceptor	Exiguobacterium sp. MH3	NC_022794.1	31009	0.167	0.09	0.067	0.000*
Acceptor	Rhodanobacter denitrificans	NC_020541.1	155	0.144	0.109	0.036	0.000*
Acceptor	Staphylococcus aureus subsp. aureus	NZ_AP014652.1	55	0.002	0.22	-0.218	-0.000*
Acceptor	Shigella dysenteriae Sd197	NC_007606.1	551	0.09	0.113	-0.023	-0.000*
Acceptor	Sanguibacter keddieii DSM 10542	NC_013521.1	54	0.003	0.247	-0.244	-0.000*
Acceptor	Kytococcus sedentarius DSM 20547	NC_013169.1	51	0.003	0.266	-0.262	-0.000*
Acceptor	Cellulomonas fimi ATCC 484	NC_015514.1	70	0.004	0.257	-0.253	-0.000*
Acceptor	Kineococcus radiotolerans SRS30216 = ATCC BAA-149	NC_009664.2	75	0.009	0.302	-0.294	-0.000*
Acceptor	Rhodobacter sp. LPB0142	NZ_CP017781.1	65	0.008	0.373	-0.365	-0.000*
Acceptor	Bacillus subtilis	NZ_LN649259.1	69	0.002	0.350	-0.348	-0.000*
Acceptor	Herbaspirillum rubrisubalbicans M1	NZ_CP013737.1	80	0.004	0.322	-0.318	-0.000*
Acceptor	Selenomonas sp. oral taxon 478	NZ_CP012071.1	67	0.002	0.41	-0.408	-0.000*
Acceptor	Serinicoccus sp. JLT9	NZ_CP014989.1	93	0.015	0.325	-0.310	-0.000*
Acceptor	Acidovorax sp. JS42	NC_008782.1	70	0.001	0.473	-0.472	-0.000*
Acceptor	Streptococcus pneumoniae CGSP14	NC_010582.1	69	0.006	0.489	-0.483	-0.000*
Acceptor	Clostridioides difficile M120	NC_017174.1	79	0.002	0.425	-0.423	-0.000*
Acceptor	Streptococcus pyogenes	NZ_CP007241.1	68	0.001	0.496	-0.495	-0.000*
Acceptor	Escherichia fergusonii ATCC 35469	NC_011740.1	83	0.009	0.417	-0.408	-0.000*
Acceptor	Herbaspirillum seropedicae	NZ_CP011930.1	117	0.007	0.31	-0.303	-0.000*
Acceptor	Streptococcus agalactiae 2603V/R	NC_004116.1	64	0.001	0.555	-0.554	-0.000*
Acceptor	Streptococcus suis	NZ_CP007497.1	66	0.002	0.542	-0.54	-0.000*
Acceptor	Collimonas arenae	NZ_CP013233.1	109	0.003	0.359	-0.356	-0.000*
Donor	Streptococcus pyogenes	NZ_CP007240.1	68	0.001	0.982	-0.980	-0.000*
Donor	Streptococcus constellatus subsp. pharyngis C1050	NC_022238.1	68	0.001	0.951	-0.950	-0.000*
Donor	Streptococcus agalactiae	NZ_CP013908.1	65	0.001	0.915	-0.914	-0.000*
Donor	Aerococcus christensenii	NZ_CP014159.1	68	0.002	0.911	-0.91	-0.000*
Donor	Rhodococcus sp. p52	NZ_CP016819.1	115	0.003	0.869	-0.866	-0.000*
Donor	Gordonia bronchialis DSM 43247	NC_013441.1	307	0.003	0.864	-0.862	-0.000*
Donor	Shinella sp. HZN7	NZ_CP015736.1	107	0.002	0.85	-0.848	-0.000*
Donor	Janthinobacterium sp. Marseille	NC_009659.1	99	0.002	0.836	-0.834	-0.000*
Donor	Nocardia soli	NZ_CP018082.1	214	0.001	0.824	-0.823	-0.000*
Donor	Herminiimonas arsenicoxydans	NC_009138.1	74	0.003	0.817	-0.815	-0.000*
Donor	Enterococcus faecium Aus0085	NC_021994.1	85	0.002	0.795	-0.793	-0.000*
**Donor**	**Helicobacter pylori**	**NZ_AP014710.1**	**925**	**0.018**	**0.804**	**-0.786**	**-0.000***
Donor	Ochrobactrum anthropi ATCC 49188	NC_009668.1	211	0.006	0.719	-0.713	-0.000*
Donor	Streptococcus suis TL13	NC_021213.1	70	0.003	0.689	-0.686	-0.000*
Donor	Erysipelothrix rhusiopathiae	NZ_CP014861.1	78	0.003	0.687	-0.684	-0.000*
Donor	Rhodococcus pyridinivorans SB3094	NC_023150.1	100	0.003	0.662	-0.659	-0.000*
Donor	Defluviimonas alba	NZ_CP012661.1	139	0.012	0.657	-0.645	-0.000*
Donor	Escherichia albertii KF1	NZ_CP007025.1	117	0.003	0.627	-0.624	-0.000*
Donor	Brevibacterium linens	NZ_CP014869.1	104	0.001	0.616	-0.615	-0.000*
Donor	Staphylococcus epidermidis RP62A	NC_002976.3	131	0.001	0.601	-0.59	-0.000*
Donor	Staphylococcus pseudintermedius HKU10-03	NC_014925.1	143	0.001	0.586	-0.585	-0.000*
Donor	Propionibacterium freudenreichii subsp. freudenreichii	NZ_CP010341.1	203	0.002	0.569	-0.567	-0.000*
Donor	Enterobacter asburiae	NZ_CP011863.1	103	0.002	0.561	-0.559	-0.000*
Donor	Pannonibacter phragmitetus	NZ_CP013068.1	144	0.001	0.552	-0.550	-0.000*
Donor	Herminiimonas sp. AS8	NZ_LT671418.1	97	0.002	0.489	-0.487	-0.000*
Donor	Streptococcus pneumoniae CGSP14	NC_010582.1	69	0.006	0.489	-0.483	-0.000*
Donor	Acidovorax sp. JS42	NC_008782.1	70	0.001	0.473	-0.472	-0.000*
Donor	Janthinobacterium agaricidamnosum NBRC 102515 = DSM 9628	NZ_HG322949.1	416	0.029	0.488	-0.459	-0.000*
Donor	Collimonas pratensis	NZ_CP013236.1	104	0.002	0.46	-0.458	-0.000*
Donor	Arsenicicoccus sp. oral taxon 190	NZ_CP012070.1	242	0.006	0.451	-0.445	-0.000*
Acceptor-like Donor	Nonlabens dokdonensis DSW-6	NC_020156.1	4139	0.976	0.014	0.961	0.000*
Acceptor-like Donor	Paracoccus denitrificans PD1222	NC_008687.1	65818	0.989	0.033	0.956	0.003
