# Supplementary material for: Where did you come from, where did you go: Refining metagenomic analysis tools for horizontal gene transfer characterisation
Source: PLoS Comput Biol. 2019 Jul 23;15(7):e1007208. doi: 10.1371/journal.pcbi.1007208 (PMC6677323; doi:10.1371/journal.pcbi.1007208)
Supplement: S10 Table — (PDF) [file pcbi.1007208.s010.pdf]

S10 Table: Acceptor and donor candidates for cami medium complexity 10% sub-sampling and *H. pylori* 10% sub-sampling run with yara, one candidate per species, no species filter and no samflag filter. Sampling sensitivity = 90. No taxon blacklist. No parent blacklist. No species blacklist. $(-)0.000^{*}$ represents absolute values $< 0.000\bar{4}$. The true positive acceptor and donor of the spiked in HGT organism are marked in bold.

Candidate			MicrobeGPS metrics			DaisyGPS metrics	
Type	Name	Accession.Version	Number Reads	Validity	Heterogeneity	Donor Score	Acceptor Score
Acceptor	Sinorhizobium meliloti AK83	NC_015590.1	128575	0.978	0.004	0.974	0.052
Acceptor	Azospirillum brasilense	NZ_CP012914.1	30150	0.976	0.007	0.968	0.012
Acceptor	Moorella thermoacetica	NZ_CP012369.1	27961	0.968	0.006	0.962	0.011
Acceptor	Exiguobacterium sp. MH3	NC_022794.1	21681	0.78	0.03	0.750	0.007
Acceptor	Acetoanaerobium sticklandii	NC_014614.1	18587	0.550	0.074	0.476	0.004
Acceptor	Streptomyces griseus subsp. griseus NBRC 13350	NC_010572.1	19509	0.311	0.085	0.227	0.002
Acceptor	Clostridium tetani E88	NC_004557.1	4093	0.965	0.018	0.948	0.002
**Acceptor**	**Escherichia coli str. K-12 substr. DH10B**	**NC_010473.1**	**19729**	**0.216**	**0.08**	**0.136**	**0.001**
Acceptor	Thermoanaerobacter wiegelii Rt8.B1	NC_015958.1	5102	0.522	0.060	0.461	0.001
Acceptor	Rhodobacter capsulatus SB 1003	NC_014034.1	2294	0.782	0.037	0.745	0.001
Acceptor	Tetragenococcus halophilus NBRC 12172	NC_016052.1	2174	0.688	0.032	0.656	0.001
Acceptor	Methylibium petroleiphilum PM1	NC_008825.1	2351	0.536	0.083	0.453	0.000*
Acceptor	Butyrivibrio hungatei	NZ_CP017831.1	938	0.838	0.031	0.808	0.000*
Acceptor	Thermoanaerobacter brockii subsp. finnii Ako-1	NC_014964.1	2996	0.285	0.073	0.212	0.000*
Acceptor	Thermoanaerobacter pseudethanolicus ATCC 33223	NC_010321.1	2998	0.284	0.076	0.209	0.000*
Acceptor	Streptomyces globisporus C-1027	NZ_CP013738.1	7491	0.157	0.079	0.078	0.000*
Acceptor	Exiguobacterium sp. U13-1	NZ_CP015731.1	4474	0.175	0.058	0.117	0.000*
Acceptor	Exiguobacterium sp. ZWU0009	NZ_CP018057.1	4319	0.167	0.055	0.112	0.000*
Acceptor	Streptomyces sp. Tue 6075	NZ_CP010833.1	6951	0.145	0.079	0.066	0.000*
Acceptor	Thermoanaerobacter sp. X513	NC_014538.1	2247	0.265	0.062	0.203	0.000*
Acceptor	Thermoanaerobacter sp. X514	NC_010320.1	2248	0.263	0.067	0.196	0.000*
Acceptor	Variovorax paradoxus S110	NC_012791.1	2209	0.252	0.106	0.146	0.000*
Acceptor	Xanthobacter autotrophicus Py2	NC_009720.1	426	0.479	0.037	0.443	0.000*
Acceptor	Nitrosomonas europaea ATCC 19718	NC_004757.1	158	0.868	0.051	0.817	0.000*
Acceptor	Acholeplasma oculi	NZ_LK028559.1	153	0.873	0.077	0.796	0.000*
Acceptor	Bacillus coagulans DSM 1 = ATCC 7050	NZ_CP009709.1	139	0.845	0.055	0.791	0.000*
Acceptor	Sphingomonas wittichii RW1	NC_009511.1	373	0.264	0.113	0.151	0.000*
Acceptor	Acidovorax sp. KKS102	NC_018708.1	1419	0.113	0.073	0.04	0.000*
Acceptor	Thermoanaerobacter kivui	NZ_CP009170.1	1458	0.176	0.139	0.037	0.000*
Acceptor	Paracoccus denitrificans PD1222	NC_008686.1	199	0.305	0.079	0.226	0.000*
Donor	Limnohabitans sp. 63ED37-2	NZ_CP011774.1	52	0.005	0.964	-0.959	-0.000*
Donor	Mahella australiensis 50-1 BON	NC_015520.1	77	0.005	0.944	-0.939	-0.000*
Donor	Hoeflea sp. IMCC20628	NZ_CP011479.1	60	0.002	0.888	-0.886	-0.000*
Donor	Myroides odoratimimus	NZ_CP013690.1	90	0.017	0.81	-0.793	-0.000*
Donor	[Clostridium] clariflavum DSM 19732	NC_016627.1	109	0.025	0.809	-0.784	-0.000*
Donor	Martelella endophytica	NZ_CP010803.1	60	0.004	0.786	-0.782	-0.000*
**Donor**	**Helicobacter pylori**	**NZ_AP014710.1**	**925**	**0.018**	**0.798**	**-0.781**	**-0.000***
Donor	Rhodoferax saidenbachensis	NZ_CP019239.1	70	0.003	0.76	-0.758	-0.000*
Donor	Brevundimonas sp. GW460-12-10-14-LB2	NZ_CP015511.1	241	0.004	0.762	-0.757	-0.000*
Donor	Sphingomonas hengshuiensis	NZ_CP010836.1	82	0.005	0.748	-0.743	-0.000*
Donor	Pelagibacterium halotolerans B2	NC_016078.1	106	0.002	0.728	-0.727	-0.000*
Donor	Martelella sp. AD-3	NZ_CP014275.1	63	0.007	0.704	-0.697	-0.000*
Donor	Rhizobium sp. NT-26	NZ_FO082820.1	117	0.004	0.699	-0.696	-0.000*
Donor	[Clostridium] stercorarium subsp. leptospartum DSM 9219	NZ_CP014673.1	61	0.011	0.682	-0.671	-0.000*
Donor	Geobacter sp. M18	NC_014973.1	51	0.013	0.658	-0.646	-0.000*
Donor	Escherichia albertii KF1	NZ_CP007025.1	117	0.003	0.627	-0.624	-0.000*
Donor	Sphingobacterium sp. ML3W	NZ_CP009278.1	55	0.005	0.628	-0.622	-0.000*
Donor	Cupriavidus basilensis	NZ_CP010537.1	59	0.002	0.620	-0.618	-0.000*
Donor	Geoalkalibacter subterraneus	NZ_CP010311.1	55	0.002	0.617	-0.615	-0.000*
Donor	Streptomyces clavuligerus	NZ_CP016559.1	50	0.006	0.613	-0.607	-0.000*
Donor	Streptomyces lividans TK24	NZ_CP009124.1	87	0.005	0.609	-0.603	-0.000*
Donor	Mesorhizobium amorphae CCNWGS0123	NZ_CP015318.1	90	0.004	0.604	-0.59	-0.000*
Donor	Defluviitoga tunisiensis	NZ_LN824141.1	52	0.015	0.587	-0.573	-0.000*
Donor	Mesorhizobium ciceri biovar biserrulae	NZ_CP015064.1	67	0.003	0.571	-0.568	-0.000*
Donor	Rhizobium gallicum bv. gallicum R602	NZ_CP006877.1	145	0.001	0.569	-0.568	-0.000*
Donor	Enterobacter asburiae	NZ_CP011863.1	103	0.002	0.561	-0.559	-0.000*
Donor	Thermoanaerobacterium thermosaccharolyticum DSM 571	NC_014410.1	55	0.014	0.572	-0.558	-0.000*
Donor	Mesorhizobium loti MAFF303099	NC_002678.2	60	0.002	0.559	-0.557	-0.000*
Donor	Sphingomonas sp. ABOJV	NZ_CP018820.1	62	0.002	0.554	-0.551	-0.000*
Donor	Rhodoferax sp. DCY110	NZ_CP019236.1	92	0.004	0.554	-0.549	-0.000*
Acceptor-like Donor	Sinorhizobium meliloti AK83	NC_015591.1	40892	0.857	0.037	0.820	0.014
Acceptor-like Donor	Nitrosomonas europaea ATCC 19718	NC_004757.1	158	0.868	0.051	0.817	0.000*
