# Supplementary material for: Where did you come from, where did you go: Refining metagenomic analysis tools for horizontal gene transfer characterisation
Source: PLoS Comput Biol. 2019 Jul 23;15(7):e1007208. doi: 10.1371/journal.pcbi.1007208 (PMC6677323; doi:10.1371/journal.pcbi.1007208)
Supplement: S11 Table — (PDF) [file pcbi.1007208.s011.pdf]

S11 Table: Acceptor and donor candidates for cami high complexity 10% sub-sampling and *H. pylori* 10% sub-sampling run with yara, one candidate per species, no species filter and no samflag filter. Sampling sensitivity = 90. No taxon blacklist. No parent blacklist. No species blacklist. (-)0.000^{*} represents absolute values $< 0.000\bar{4}$. The true positive acceptor and donor of the spiked in HGT organism are marked in bold.

Candidate			MicrobeGPS metrics			DaisyGPS metrics	
Type	Name	Accession.Version	Number Reads	Validity	Heterogeneity	Donor Score	Acceptor Score
Acceptor	Desulfococcus multivorans	NZ_CP015381.1	42454	0.861	0.022	0.839	0.021
Acceptor	Desulfovibrio alaskensis G20	NC_007519.1	28439	0.881	0.021	0.86	0.015
Acceptor	Ensifer adhaerens	NZ_CP015880.1	20775	0.907	0.015	0.892	0.011
Acceptor	Sphingomonas sanxanigenens DSM 19645 = NX02	NZ_CP006644.1	16536	0.973	0.007	0.967	0.01
Acceptor	Microbacterium sp. BH3-3-3	NZ_CP017674.1	13323	0.868	0.02	0.848	0.007
Acceptor	Actinosynnema mirum DSM 43827	NC_013093.1	22642	0.498	0.072	0.426	0.006
Acceptor	Agrobacterium sp. H13-3	NC_015183.1	9986	0.960	0.013	0.947	0.006
Acceptor	Agrobacterium tumefaciens	NZ_CP011246.1	9687	0.958	0.022	0.937	0.005
Acceptor	Pseudomonas fluorescens	NZ_CP011117.1	8323	0.883	0.033	0.85	0.004
Acceptor	Methylobacterium extorquens AM1	NC_012808.1	8404	0.854	0.033	0.821	0.004
Acceptor	Rhodococcus sp. PBTS 2	NZ_CP015220.1	6540	0.897	0.043	0.854	0.003
Acceptor	Rhodococcus fascians D188	NZ_CP015235.1	6509	0.876	0.041	0.836	0.003
Acceptor	Methylobacterium radiotolerans JCM 2831	NC_010505.1	5690	0.734	0.051	0.683	0.002
Acceptor	Methylobacterium sp. C1	NZ_CP017640.1	5686	0.721	0.071	0.65	0.002
Acceptor	Pseudomonas brassicacearum subsp. brassicacearum NFM421	NC_015379.1	4453	0.847	0.034	0.813	0.002
Acceptor	Microbacterium sp. 1.5R	NZ_CP018151.1	3756	0.880	0.027	0.853	0.002
Acceptor	Microbacterium sp. CGR1	NZ_CP012299.1	3561	0.877	0.028	0.849	0.002
**Acceptor**	**Escherichia coli str. K-12 substr. DH10B**	**NC_010473.1**	**19730**	**0.216**	**0.08**	**0.136**	**0.002**
Acceptor	Pseudomonas koreensis	NZ_CP014947.1	3029	0.875	0.033	0.843	0.002
Acceptor	Pseudomonas frederiksbergensis	NZ_CP018319.1	4273	0.689	0.092	0.597	0.002
Acceptor	Bacillus pumilus	NZ_AP014928.1	2763	0.894	0.031	0.863	0.001
Acceptor	Bacillus megaterium QM B1551	NC_014019.1	2740	0.79	0.097	0.703	0.001
Acceptor	Bacillus sp. IHB B 7164	NZ_CP015226.1	2633	0.824	0.104	0.720	0.001
Acceptor	Methylobacterium sp. AMS5	NZ_CP006992.1	2605	0.712	0.034	0.677	0.001
Acceptor	Bacillus safensis	NZ_CP018197.1	1767	0.905	0.033	0.872	0.001
Acceptor	Acidovorax sp. KKS102	NC_018708.1	3023	0.551	0.054	0.497	0.001
Acceptor	Pseudarthrobacter chlorophenolicus A6	NC_011886.1	8107	0.247	0.088	0.159	0.001
Acceptor	Desulfitobacterium hafniense Y51	NC_007907.1	2394	0.603	0.068	0.535	0.001
Acceptor	Azotobacter chroococcum NCIMB 8003	NZ_CP010415.1	4889	0.303	0.047	0.256	0.001
Acceptor	Elizabethkingia miricola	NZ_CP011059.1	2211	0.508	0.061	0.448	0.001
Donor	Streptococcus pyogenes	NZ_CP007240.1	70	0.01	0.982	-0.972	-0.000*
Donor	Proteus vulgaris	NZ_CP012675.1	88	0.003	0.957	-0.954	-0.000*
Donor	Proteus mirabilis	NZ_CP012674.1	88	0.003	0.957	-0.954	-0.000*
Donor	Streptococcus pneumoniae TCH8431/19A	NC_014251.1	62	0.007	0.96	-0.953	-0.000*
Donor	Vibrio cholerae O1 str. 2010EL-1786	NC_016445.1	131	0.003	0.951	-0.948	-0.000*
Donor	Listeria monocytogenes ATCC 19117	NZ_CP013288.1	52	0.006	0.951	-0.945	-0.000*
Donor	Streptococcus oralis Uo5	NC_015291.1	67	0.008	0.932	-0.924	-0.000*
Donor	Dehalogenimonas lykanthroporepellens BL-DC-9	NC_014314.1	152	0.029	0.952	-0.923	-0.000*
Donor	Syntrophobacter fumaroxidans MPOB	NC_008554.1	218	0.013	0.922	-0.909	-0.000*
Donor	Streptococcus agalactiae	NZ_CP013908.1	70	0.008	0.908	-0.901	-0.000*
Donor	Aerococcus christensenii	NZ_CP014159.1	68	0.01	0.902	-0.893	-0.000*
Donor	Hafnia alvei FB1	NZ_CP009706.1	87	0.002	0.886	-0.883	-0.000*
Donor	Streptococcus constellatus subsp. pharyngis C1050	NC_022238.1	71	0.01	0.889	-0.88	-0.000*
Donor	Steroidobacter denitrificans	NZ_CP011971.1	55	0.004	0.872	-0.868	-0.000*
Donor	Streptococcus pseudopneumoniae IS7493	NC_015875.1	67	0.007	0.873	-0.865	-0.000*
Donor	Edwardsiella tarda FL6-60	NC_017309.1	59	0.003	0.863	-0.861	-0.000*
Donor	Pseudomonas aeruginosa	NZ_CP007399.1	65	0.009	0.853	-0.845	-0.000*
Donor	Streptococcus anginosus	NZ_CP012805.1	70	0.008	0.852	-0.844	-0.000*
Donor	Chelativorans sp. BNC1	NC_008254.1	64	0.009	0.848	-0.839	-0.000*
Donor	Staphylococcus pseudintermedius HKU10-03	NC_014925.1	67	0.006	0.839	-0.833	-0.000*
Donor	Providencia stuartii	NZ_CP008920.1	88	0.003	0.832	-0.829	-0.000*
Donor	Dichelobacter nodosus VCS1703A	NC_009446.1	50	0.008	0.831	-0.823	-0.000*
Donor	Sphingobium japonicum UT26S	NC_014006.1	296	0.022	0.828	-0.806	-0.000*
**Donor**	**Helicobacter pylori**	**NZ_AP014710.1**	**925**	**0.018**	**0.804**	**-0.786**	**-0.000***
Donor	Anaerococcus sp. Marseille-P2765	NZ_LT635772.1	59	0.009	0.789	-0.78	-0.000*
Donor	Vibrio parahaemolyticus	NZ_CP010883.1	133	0.001	0.78	-0.778	-0.000*
Donor	Lactobacillus johnsonii	NZ_CP016400.1	76	0.009	0.782	-0.773	-0.000*
Donor	Enterococcus faecium Aus0085	NC_021994.1	72	0.007	0.766	-0.759	-0.000*
Donor	Streptococcus lutetiensis 033	NC_021900.1	70	0.009	0.761	-0.752	-0.000*
Donor	Streptococcus gordonii	NZ_CP012648.1	71	0.007	0.754	-0.747	-0.000*
Acceptor-like Donor	Sphingomonas sanxanigenens DSM 19645 = NX02	NZ_CP006644.1	16536	0.973	0.007	0.967	0.01
Acceptor-like Donor	Ensifer adhaerens	NZ_CP015880.1	20775	0.907	0.015	0.892	0.011
