# Supplementary material for: Where did you come from, where did you go: Refining metagenomic analysis tools for horizontal gene transfer characterisation
Source: PLoS Comput Biol. 2019 Jul 23;15(7):e1007208. doi: 10.1371/journal.pcbi.1007208 (PMC6677323; doi:10.1371/journal.pcbi.1007208)
Supplement: S12 Table — (PDF) [file pcbi.1007208.s012.pdf]

S12 Table: Acceptor and donor candidates for ERR103401 run with yara, species filter and no samflag filter. No taxon blacklist. No parent blacklist. No species blacklist. (-)0.000^{*} represents absolute values $< 0.000\bar{4}$. The supposed acceptor is marked in bold.

Candidate			MicrobeGPS metrics			DaisyGPS metrics	
Type	Name	Accession.Version	Number Reads	Validity	Heterogeneity	Donor Score	Acceptor Score
**Acceptor**	**Staphylococcus aureus subsp. aureus HO 5096 0412**	**NC_017763.1**	**440076**	**0.832**	**0.04**	**0.792**	**0.041**
Acceptor	Staphylococcus aureus subsp. aureus	NZ_CP007659.1	439586	0.824	0.041	0.783	0.040
Donor	Staphylococcus pseudintermedius ED99	NC_017568.1	1089	0.002	0.691	-0.689	-0.000^{*}
Donor	Staphylococcus warneri SG1	NC_020164.1	523	0.003	0.631	-0.628	-0.000^{*}
Donor	Staphylococcus epidermidis RP62A	NC_002976.3	5512	0.006	0.540	-0.534	-0.000^{*}
Donor	Staphylococcus haemolyticus JCSC1435	NC_007168.1	3614	0.005	0.291	-0.285	-0.000^{*}
Donor	Staphylococcus aureus subsp. aureus COL	NC_002951.2	49889	0.106	0.233	-0.127	-0.001
Acceptor-like Donor	Staphylococcus aureus subsp. aureus	NZ_CP012011.1	54992	0.11	0.109	0.001	0.000^{*}
