# Supplementary material for: Where did you come from, where did you go: Refining metagenomic analysis tools for horizontal gene transfer characterisation
Source: PLoS Comput Biol. 2019 Jul 23;15(7):e1007208. doi: 10.1371/journal.pcbi.1007208 (PMC6677323; doi:10.1371/journal.pcbi.1007208)
Supplement: S13 Table — (PDF) [file pcbi.1007208.s013.pdf]

S13 Table: Results for ERR103401 run with yara, gustaf, species filter and no samflag filter. Sampling sensitivity = 90. Split read threshold = 3. No taxon blacklist. No parent blacklist. No species blacklist.

Organism		Acceptor			Donor			Read Evidence			Evidence Filter			
Acceptor	Donor	Start	End	Coverage	Start	End	Coverage	Split	Spanning	Within	A-Cov	D-Cov	Spanning	Within
NZ_CP007659.1	NC_020164.1	37045	37048	413.0	121379	123703	36.08	5	36	123	100	100	100	100
NZ_CP007659.1	NC_020164.1	37045	37176	220.86	111790	121379	2.26	20	7	47	100	99	100	100
NZ_CP007659.1	NC_020164.1	37047	37125	272.67	121461	123702	24.58	7	36	103	100	100	100	100
NZ_CP007659.1	NC_020164.1	37047	37176	217.84	111790	123702	8.84	19	38	160	100	100	100	100
NZ_CP007659.1	NC_020164.1	37124	37176	134.96	111790	121460	5.17	22	7	72	100	100	98	100
NC_017763.1	NZ_CP012011.1	1525462	1554768	130.8	1228987	1251487	14.6	4	26	826	100	97	100	97
NC_017763.1	NZ_CP012011.1	1525488	1554768	130.8	1228987	1251477	14.59	10	26	826	100	100	100	100
NC_017763.1	NC_020164.1	37044	37047	412.0	121379	123703	36.18	5	36	124	100	100	100	100
NC_017763.1	NC_020164.1	37044	37175	220.35	111790	121379	2.26	20	7	47	100	100	100	100
NC_017763.1	NC_020164.1	37046	37124	271.83	121461	123702	24.69	7	36	104	100	100	100	100
NC_017763.1	NC_020164.1	37046	37175	217.34	111790	123702	8.86	19	38	161	100	100	100	100
NC_017763.1	NC_020164.1	37123	37175	134.96	111790	121460	5.17	22	7	72	100	100	100	100
NZ_CP007659.1	NC_002951.2	1568261	1575973	129.75	359692	369382	5.66	9	3	42	98	98	99	95
NZ_CP007659.1	NC_002951.2	1568261	1576904	131.71	358442	369382	6.99	41	5	87	97	97	100	98
NZ_CP007659.1	NC_002951.2	1568261	1579141	126.48	356047	369382	11.01	5	3	264	99	99	100	99
NZ_CP007659.1	NC_002951.2	1568286	1575973	129.72	359692	369358	5.64	7	3	42	100	99	100	97
NZ_CP007659.1	NC_002951.2	1568286	1576904	131.69	358442	369358	6.97	39	5	87	100	98	100	98
NZ_CP007659.1	NC_002951.2	1568286	1579141	126.45	356047	369358	11.0	3	3	264	97	98	100	98
NZ_CP007659.1	NC_002951.2	1568948	1575973	132.04	359692	369170	5.6	6	1	40	100	99	99	96
NZ_CP007659.1	NC_002951.2	1568948	1576904	133.9	358442	369170	6.95	22	3	85	100	95	100	97
NZ_CP007659.1	NC_002951.2	1568948	1579141	127.84	356047	369170	11.04	4	1	262	100	99	98	97
NZ_CP007659.1	NC_002951.2	1575972	1576904	147.92	358442	359691	17.26	58	2	45	100	98	100	99
NZ_CP007659.1	NC_002951.2	1576903	1579141	106.26	356047	358441	29.37	13	29	177	94	99	100	100
NC_017763.1	NC_002976.3	37130	37175	108.33	2256184	2258869	26.44	25	206	76	92	99	100	99
NZ_CP007659.1	NZ_CP012011.1	1539648	1568954	130.85	1228987	1251487	14.6	4	26	826	100	94	99	93
NZ_CP007659.1	NZ_CP012011.1	1539674	1568954	130.85	1228987	1251477	14.59	10	26	826	100	97	100	97
NC_017763.1	NC_002951.2	1554075	1561787	129.75	359692	369382	5.66	9	3	42	99	98	100	94
NC_017763.1	NC_002951.2	1554075	1562718	131.71	358442	369382	6.99	41	5	87	100	96	100	97
NC_017763.1	NC_002951.2	1554075	1564955	126.48	356047	369382	11.01	5	3	264	97	97	100	97
NC_017763.1	NC_002951.2	1554100	1561787	129.72	359692	369358	5.64	7	3	42	98	99	99	94
NC_017763.1	NC_002951.2	1554100	1562718	131.69	358442	369358	6.97	39	5	87	99	96	100	98
NC_017763.1	NC_002951.2	1554100	1564955	126.45	356047	369358	11.0	3	3	264	99	99	99	99
NC_017763.1	NC_002951.2	1554762	1561787	132.04	359692	369170	5.6	6	1	40	99	100	100	96
NC_017763.1	NC_002951.2	1554762	1562718	133.9	358442	369170	6.95	22	3	85	99	98	99	97
NC_017763.1	NC_002951.2	1554762	1564955	127.84	356047	369170	11.04	4	1	262	98	97	99	97
NC_017763.1	NC_002951.2	1561786	1562718	147.92	358442	359691	17.26	58	2	45	100	97	100	98
NC_017763.1	NC_002951.2	1562717	1564955	106.26	356047	358441	29.37	13	29	177	95	98	100	99
NZ_CP007659.1	NC_002976.3	37131	37176	108.33	2256184	2258869	26.44	25	206	76	93	100	100	100
