# Supplementary material for: Where did you come from, where did you go: Refining metagenomic analysis tools for horizontal gene transfer characterisation
Source: PLoS Comput Biol. 2019 Jul 23;15(7):e1007208. doi: 10.1371/journal.pcbi.1007208 (PMC6677323; doi:10.1371/journal.pcbi.1007208)
Supplement: S14 Table — (PDF) [file pcbi.1007208.s014.pdf]

S14 Table: Acceptor and donor candidates for ERR103403 run with yara, species filter and no samflag filter. Sampling sensitivity = 85. No taxon blacklist. No parent blacklist. No species blacklist. (-)0.000^{*} represents absolute values $< 0.000\bar{4}$. The supposed acceptor is marked in bold.

Candidate			MicrobeGPS metrics			DaisyGPS metrics	
Type	Name	Accession.Version	Number Reads	Validity	Heterogeneity	Donor Score	Acceptor Score
**Acceptor**	**Staphylococcus aureus subsp. aureus HO 5096 0412**	**NC_017763.1**	**206493**	**0.813**	**0.063**	**0.750**	**0.039**
Acceptor	Staphylococcus aureus subsp. aureus	NZ_CP007659.1	206231	0.806	0.066	0.74	0.038
Donor	Staphylococcus warneri SG1	NC_020164.1	196	0.003	0.639	-0.636	-0.000*
Donor	Staphylococcus pseudintermedius HKU10-03	NC_014925.1	705	0.001	0.582	-0.581	-0.000*
Donor	Staphylococcus epidermidis RP62A	NC_002976.3	2171	0.005	0.537	-0.532	-0.000*
Donor	Staphylococcus haemolyticus JCSC1435	NC_007168.1	1398	0.005	0.287	-0.283	-0.000*
Donor	Staphylococcus aureus subsp. aureus TW20	NC_017331.1	27837	0.096	0.364	-0.268	-0.002
Acceptor-like Donor	Staphylococcus aureus CA-347	NC_021554.1	31231	0.148	0.146	0.003	0.000*
