# Supplementary material for: Where did you come from, where did you go: Refining metagenomic analysis tools for horizontal gene transfer characterisation
Source: PLoS Comput Biol. 2019 Jul 23;15(7):e1007208. doi: 10.1371/journal.pcbi.1007208 (PMC6677323; doi:10.1371/journal.pcbi.1007208)
Supplement: S15 Table — (PDF) [file pcbi.1007208.s015.pdf]

S15 Table: Results for ERR103403 run with yara, gustaf, species filter and no samflag filter. Sampling sensitivity = 90. Split read threshold = 3. No taxon blacklist. No parent blacklist. No species blacklist.

Organism		Acceptor			Donor			Read Evidence			Evidence Filter			
Acceptor	Donor	Start	End	Coverage	Start	End	Coverage	Split	Spanning	Within	A-Cov	D-Cov	Spanning	Within
NZ_CP007659.1	NC_021554.1	1568897	1578954	57.77	1567257	1577035	1.38	3	4	35	98	97	100	97
NC_017763.1	NC_017331.1	1525080	1525467	58.54	413136	417103	24.3	11	6	302	96	98	100	99
NC_017763.1	NC_017331.1	1525080	1525489	59.9	413114	417103	24.24	17	8	302	99	100	100	100
NC_017763.1	NC_017331.1	1525080	1559823	62.98	382945	417103	15.53	6	7	1608	99	99	98	100
NC_017763.1	NC_017331.1	1525466	1559823	63.03	382945	413135	14.37	5	18	1306	100	100	99	100
NC_017763.1	NC_017331.1	1525466	1561786	62.78	381925	413135	13.92	25	11	1306	98	97	100	100
NC_017763.1	NC_017331.1	1525488	1559823	63.02	382945	413113	14.37	13	18	1306	99	100	100	100
NC_017763.1	NC_017331.1	1525488	1561786	62.77	381925	413113	13.92	23	11	1306	97	99	99	100
NC_017763.1	NC_014925.1	36951	37132	244.55	906205	906387	96.3	26	10	11	100	100	100	100
NC_017763.1	NC_014925.1	36951	37151	233.42	906205	906409	93.52	20	10	11	96	100	100	100
NC_017763.1	NC_014925.1	37044	37151	163.06	906300	906409	116.82	4	9	11	100	100	100	100
NZ_CP007659.1	NC_014925.1	36952	37133	244.55	906205	906387	96.3	26	10	11	100	100	100	100
NZ_CP007659.1	NC_014925.1	36952	37152	233.42	906205	906409	93.52	20	10	11	100	100	100	100
NZ_CP007659.1	NC_014925.1	37045	37152	163.06	906300	906409	116.82	4	9	11	100	100	100	100
NZ_CP007659.1	NC_017331.1	1539266	1539653	58.54	413136	417103	24.3	11	6	302	98	98	100	99
NZ_CP007659.1	NC_017331.1	1539266	1539675	59.9	413114	417103	24.24	17	8	302	100	100	100	100
NZ_CP007659.1	NC_017331.1	1539266	1574009	62.99	382945	417103	15.53	6	7	1608	100	100	97	100
NZ_CP007659.1	NC_017331.1	1539652	1574009	63.04	382945	413135	14.37	5	18	1306	99	100	100	100
NZ_CP007659.1	NC_017331.1	1539652	1575972	62.79	381925	413135	13.92	25	11	1306	95	98	99	100
NZ_CP007659.1	NC_017331.1	1539674	1574009	63.02	382945	413113	14.37	13	18	1306	98	99	100	100
NZ_CP007659.1	NC_017331.1	1539674	1575972	62.77	381925	413113	13.92	23	11	1306	99	99	99	100
NC_017763.1	NC_021554.1	1554711	1564768	57.77	1567257	1577035	1.38	3	4	35	100	98	99	98
