# Supplementary material for: Where did you come from, where did you go: Refining metagenomic analysis tools for horizontal gene transfer characterisation
Source: PLoS Comput Biol. 2019 Jul 23;15(7):e1007208. doi: 10.1371/journal.pcbi.1007208 (PMC6677323; doi:10.1371/journal.pcbi.1007208)
Supplement: S16 Table — (PDF) [file pcbi.1007208.s016.pdf]

S16 Table: Acceptor and donor candidates for ERR103404 run with yara, species filter and no samflag filter. Sampling sensitivity = 85. No taxon blacklist. No parent blacklist. No species blacklist. (-)0.000^* represents absolute values $< 0.000\bar{4}$. The supposed acceptor is marked in bold.

Candidate			MicrobeGPS metrics			DaisyGPS metrics	
Type	Name	Accession.Version	Number Reads	Validity	Heterogeneity	Donor Score	Acceptor Score
**Acceptor**	**Staphylococcus aureus subsp. aureus HO 5096 0412**	**NC_017763.1**	**193345**	**0.812**	**0.043**	**0.769**	**0.041**
Acceptor	Staphylococcus aureus subsp. aureus	NZ_CP007659.1	193065	0.805	0.044	0.761	0.041
Donor	Staphylococcus pseudintermedius ED99	NC_017568.1	459	0.001	0.702	-0.700	-0.000*
Donor	Staphylococcus warneri SG1	NC_020164.1	244	0.003	0.631	-0.627	-0.000*
Donor	Staphylococcus epidermidis RP62A	NC_002976.3	2256	0.005	0.536	-0.531	-0.000*
Donor	Staphylococcus haemolyticus JCSC1435	NC_007168.1	1441	0.005	0.299	-0.295	-0.000*
Donor	Staphylococcus aureus subsp. aureus COL	NC_002951.2	20891	0.101	0.233	-0.133	-0.001
Acceptor-like Donor	Staphylococcus aureus subsp. aureus DSM 20231	NZ_CP011526.1	16400	0.102	0.084	0.018	0.000*
