# Supplementary material for: Where did you come from, where did you go: Refining metagenomic analysis tools for horizontal gene transfer characterisation
Source: PLoS Comput Biol. 2019 Jul 23;15(7):e1007208. doi: 10.1371/journal.pcbi.1007208 (PMC6677323; doi:10.1371/journal.pcbi.1007208)
Supplement: S17 Table — (PDF) [file pcbi.1007208.s017.pdf]

S17 Table: Results for ERR103404 run with yara, gustaf, species filter and no samflag filter. Sampling sensitivity = 90. Split read threshold = 3. No taxon blacklist. No parent blacklist. No species blacklist.

Organism		Acceptor			Donor			Read Evidence			Evidence Filter			
Acceptor	Donor	Start	End	Coverage	Start	End	Coverage	Split	Spanning	Within	A-Cov	D-Cov	Spanning	Within
NC_017763.1	NZ_CP011526.1	1554767	1561786	52.96	846400	854250	11.47	24	1	254	100	100	100	100
NZ_CP007659.1	NZ_CP011526.1	1568953	1575972	52.96	846400	854250	11.47	24	1	254	100	100	100	100
NZ_CP007659.1	NC_002951.2	1568275	1575973	50.88	359692	369368	2.48	7	1	24	98	98	100	95
NZ_CP007659.1	NC_002951.2	1568275	1576904	52.23	358442	369368	2.96	23	2	43	98	98	100	98
NZ_CP007659.1	NC_002951.2	1575972	1576904	63.34	358442	359691	6.72	34	1	19	100	100	100	100
NC_017763.1	NC_002951.2	1554089	1561787	50.88	359692	369368	2.48	7	1	24	98	98	100	94
NC_017763.1	NC_002951.2	1554089	1562718	52.23	358442	369368	2.96	23	2	43	100	98	100	99
NC_017763.1	NC_002951.2	1561786	1562718	63.34	358442	359691	6.72	34	1	19	100	98	100	100
NC_017763.1	NC_002951.2	2045963	2074149	30.15	369125	397269	12.8	12	10	330	6	100	97	100
