# Supplementary material for: Where did you come from, where did you go: Refining metagenomic analysis tools for horizontal gene transfer characterisation
Source: PLoS Comput Biol. 2019 Jul 23;15(7):e1007208. doi: 10.1371/journal.pcbi.1007208 (PMC6677323; doi:10.1371/journal.pcbi.1007208)
Supplement: S18 Table — (PDF) [file pcbi.1007208.s018.pdf]

S18 Table: Acceptor and donor candidates for ERR103405 run with yara, species filter and no samflag filter. Sampling sensitivity = 85. No taxon blacklist. No parent blacklist. No species blacklist. (-)0.000^* represents absolute values $< 0.000\bar{4}$. The supposed acceptor is marked in bold.

Candidate			MicrobeGPS metrics			DaisyGPS metrics	
Type	Name	Accession.Version	Number Reads	Validity	Heterogeneity	Donor Score	Acceptor Score
**Acceptor**	**Staphylococcus aureus subsp. aureus HO 5096 0412**	**NC_017763.1**	**192851**	**0.811**	**0.03**	**0.781**	**0.041**
Acceptor	Staphylococcus aureus subsp. aureus	NZ_CP007659.1	192626	0.804	0.031	0.773	0.040
Donor	Staphylococcus pseudintermedius ED99	NC_017568.1	459	0.001	0.698	-0.696	-0.000*
Donor	Staphylococcus warneri SG1	NC_020164.1	236	0.003	0.658	-0.655	-0.000*
Donor	Staphylococcus epidermidis RP62A	NC_002976.3	2006	0.005	0.543	-0.538	-0.000*
Donor	Staphylococcus haemolyticus JCSC1435	NC_007168.1	1278	0.005	0.293	-0.289	-0.000*
Donor	Staphylococcus aureus subsp. aureus COL	NC_002951.2	21599	0.097	0.227	-0.13	-0.001
Acceptor-like Donor	Staphylococcus aureus subsp. aureus	NZ_CP018205.1	20618	0.100	0.091	0.009	0.000*
