# Supplementary material for: Where did you come from, where did you go: Refining metagenomic analysis tools for horizontal gene transfer characterisation
Source: PLoS Comput Biol. 2019 Jul 23;15(7):e1007208. doi: 10.1371/journal.pcbi.1007208 (PMC6677323; doi:10.1371/journal.pcbi.1007208)
Supplement: S19 Table — (PDF) [file pcbi.1007208.s019.pdf]

S19 Table: Results for ERR103405 run with yara, gustaf, species filter and no samflag filter. Sampling sensitivity = 90. Split read threshold = 3. No taxon blacklist. No parent blacklist. No species blacklist.

Organism		Acceptor			Donor			Read Evidence			Evidence Filter			
Acceptor	Donor	Start	End	Coverage	Start	End	Coverage	Split	Spanning	Within	A-Cov	D-Cov	Spanning	Within
NC_017763.1	NZ_CP018205.1	1559883	1562718	58.73	1959491	1961823	7.95	12	1	38	100	100	100	100
NC_017763.1	NZ_CP018205.1	1561784	1562718	66.49	1960572	1961823	9.45	66	1	31	100	100	100	100
NZ_CP007659.1	NZ_CP018205.1	1574069	1576904	58.73	1959491	1961823	7.95	12	1	38	99	99	100	98
NZ_CP007659.1	NZ_CP018205.1	1575970	1576904	66.49	1960572	1961823	9.45	66	1	31	100	100	100	100
NZ_CP007659.1	NC_002951.2	1568261	1576904	56.11	358442	369382	3.13	10	1	50	100	98	100	100
NZ_CP007659.1	NC_002951.2	1575976	1576904	66.66	358442	359692	9.25	19	1	29	100	99	100	99
NZ_CP007659.1	NC_002951.2	2059982	2087935	30.68	369359	397269	12.87	5	12	341	10	100	100	100
NZ_CP007659.1	NC_002951.2	2059982	2088169	30.68	369125	397269	12.79	21	12	341	6	100	98	100
NC_017763.1	NC_002951.2	1554075	1562718	56.11	358442	369382	3.13	10	1	50	100	99	100	100
NC_017763.1	NC_002951.2	1561790	1562718	66.66	358442	359692	9.25	19	1	29	100	100	100	100
NC_017763.1	NC_002951.2	2045963	2073915	30.7	369359	397269	13.02	5	12	355	6	100	99	100
NC_017763.1	NC_002951.2	2045963	2074149	30.7	369125	397269	12.94	21	12	355	8	100	97	100
