# Supplementary material for: Where did you come from, where did you go: Refining metagenomic analysis tools for horizontal gene transfer characterisation
Source: PLoS Comput Biol. 2019 Jul 23;15(7):e1007208. doi: 10.1371/journal.pcbi.1007208 (PMC6677323; doi:10.1371/journal.pcbi.1007208)
Supplement: S20 Table — (PDF) [file pcbi.1007208.s020.pdf]

S20 Table: Acceptor and donor candidates for ERR101899 run with yara, species filter and no samflag filter. Sampling sensitivity = 85. No taxon blacklist. No parent blacklist. No species blacklist. (-)0.000^* represents absolute values $< 0.000\bar{4}$. The supposed acceptor is marked in bold.

**Candidate**			**MicrobeGPS metrics**			**DaisyGPS metrics**	
Type	Name	Accession.Version	Number Reads	Validity	Heterogeneity	Donor Score	Acceptor Score
**Acceptor**	**Staphylococcus aureus subsp. aureus HO 5096 0412**	**NC_017763.1**	**206272**	**0.814**	**0.047**	**0.767**	**0.040**
Acceptor	Staphylococcus aureus subsp. aureus	NZ_CP007659.1	206076	0.807	0.049	0.759	0.04
Donor	Staphylococcus pseudintermedius ED99	NC_017568.1	536	0.001	0.707	-0.705	-0.000^*
Donor	Staphylococcus warneri SG1	NC_020164.1	263	0.003	0.658	-0.655	-0.000^*
Donor	Staphylococcus epidermidis RP62A	NC_002976.3	2226	0.005	0.537	-0.532	-0.000^*
Donor	Staphylococcus haemolyticus JCSC1435	NC_007168.1	1378	0.004	0.296	-0.291	-0.000^*
Donor	Staphylococcus aureus subsp. aureus COL	NC_002951.2	22973	0.098	0.236	-0.139	-0.001
Acceptor-like Donor	Staphylococcus aureus subsp. aureus DSM 20231	NZ_CP011526.1	18223	0.099	0.085	0.014	0.000^*
