# Supplementary material for: Where did you come from, where did you go: Refining metagenomic analysis tools for horizontal gene transfer characterisation
Source: PLoS Comput Biol. 2019 Jul 23;15(7):e1007208. doi: 10.1371/journal.pcbi.1007208 (PMC6677323; doi:10.1371/journal.pcbi.1007208)
Supplement: S21 Table — (PDF) [file pcbi.1007208.s021.pdf]

S21 Table: Results for ERR101899 run with yara, gustaf, species filter and no samflag filter. Sampling sensitivity = 90. Split read threshold = 3. No taxon blacklist. No parent blacklist. No species blacklist.

Organism		Acceptor			Donor			Read Evidence			Evidence Filter			
Acceptor	Donor	Start	End	Coverage	Start	End	Coverage	Split	Spanning	Within	A-Cov	D-Cov	Spanning	Within
NZ_CP007659.1	NC_002951.2	1568261	1575972	53.51	359694	369382	2.62	3	2	15	99	100	100	97
NZ_CP007659.1	NC_002951.2	1568261	1576904	55.07	358442	369382	3.05	8	2	34	99	99	99	99
NZ_CP007659.1	NC_002951.2	1568287	1575972	53.49	359694	369357	2.61	3	2	15	98	100	99	99
NZ_CP007659.1	NC_002951.2	1568287	1576904	55.06	358442	369357	3.04	8	2	34	100	100	99	99
NZ_CP007659.1	NC_002951.2	2059982	2087936	31.07	369358	397269	13.23	9	15	395	10	100	100	100
NZ_CP007659.1	NC_002951.2	2059982	2088169	31.08	369125	397269	13.15	31	15	396	7	100	94	100
NZ_CP007659.1	NC_020164.1	37045	37177	87.31	111789	121379	0.85	4	2	20	100	97	100	100
NZ_CP007659.1	NZ_CP011526.1	1568903	1575972	56.42	846397	854374	12.62	9	1	267	100	100	100	100
NC_017763.1	NZ_CP011526.1	1554717	1561786	56.42	846397	854374	12.62	9	1	267	98	100	100	100
NC_017763.1	NC_002951.2	1554075	1561786	53.51	359694	369382	2.62	3	2	15	97	98	100	94
NC_017763.1	NC_002951.2	1554075	1562718	55.07	358442	369382	3.05	8	2	34	99	99	100	100
NC_017763.1	NC_002951.2	1554101	1561786	53.49	359694	369357	2.61	3	2	15	98	99	99	93
NC_017763.1	NC_002951.2	1554101	1562718	55.06	358442	369357	3.04	8	2	34	99	98	100	97
NC_017763.1	NC_002951.2	2045963	2073916	31.1	369358	397269	13.45	9	15	415	8	100	91	100
NC_017763.1	NC_020164.1	37044	37176	87.31	111789	121379	0.85	4	2	20	100	100	99	100
