# Supplementary material for: Where did you come from, where did you go: Refining metagenomic analysis tools for horizontal gene transfer characterisation
Source: PLoS Comput Biol. 2019 Jul 23;15(7):e1007208. doi: 10.1371/journal.pcbi.1007208 (PMC6677323; doi:10.1371/journal.pcbi.1007208)
Supplement: S22 Table — (PDF) [file pcbi.1007208.s022.pdf]

S22 Table: Acceptor and donor candidates for ERR101900 run with yara, species filter and no samflag filter. Sampling sensitivity = 85. No taxon blacklist. No parent blacklist. No species blacklist. (-)0.000^{*} represents absolute values $< 0.000\bar{4}$.

Candidate			MicrobeGPS metrics			DaisyGPS metrics	
Type	Name	Accession.Version	Number Reads	Validity	Heterogeneity	Donor Score	Acceptor Score
Acceptor	Staphylococcus aureus subsp. aureus	NZ_CP007659.1	162328	0.794	0.050	0.744	0.039
Donor	Staphylococcus pseudintermedius ED99	NC_017568.1	1521	0.002	0.706	-0.704	-0.000*
Donor	Staphylococcus warneri SG1	NC_020164.1	215	0.004	0.654	-0.650	-0.000*
Donor	Staphylococcus epidermidis RP62A	NC_002976.3	3028	0.005	0.560	-0.555	-0.001
Donor	Staphylococcus lugdunensis HKU09-01	NC_013893.1	53	0.002	0.358	-0.356	-0.000*
Donor	Staphylococcus haemolyticus JCSC1435	NC_007168.1	1116	0.005	0.254	-0.25	-0.000*
Donor	Staphylococcus aureus subsp. aureus COL	NC_002951.2	17868	0.103	0.242	-0.139	-0.001
Acceptor-like Donor	Staphylococcus aureus subsp. aureus NCTC 8325	NC_007795.1	16873	0.107	0.089	0.018	0.000*
