# Supplementary material for: Where did you come from, where did you go: Refining metagenomic analysis tools for horizontal gene transfer characterisation
Source: PLoS Comput Biol. 2019 Jul 23;15(7):e1007208. doi: 10.1371/journal.pcbi.1007208 (PMC6677323; doi:10.1371/journal.pcbi.1007208)
Supplement: S23 Table — (PDF) [file pcbi.1007208.s023.pdf]

S23 Table: Results for ERR101900 run with yara, gustaf, species filter and no samflag filter. Sampling sensitivity = 90. Split read threshold = 3. No taxon blacklist. No parent blacklist. No species blacklist.

Organism		Acceptor			Donor			Read Evidence			Evidence Filter			
Acceptor	Donor	Start	End	Coverage	Start	End	Coverage	Split	Spanning	Within	A-Cov	D-Cov	Spanning	Within
NC_017763.1	NC_002951.2	1554089	1562718	45.31	358442	369368	2.81	15	1	31	100	100	99	98
NC_017763.1	NC_002951.2	1554762	1562718	46.77	358442	369170	2.78	8	1	29	100	100	98	98
NC_017763.1	NC_002951.2	1561790	1562718	53.62	358442	359696	5.56	8	1	15	98	99	100	100
NZ_CP007659.1	NC_007795.1	1575971	1576904	53.59	1961777	1963027	6.06	57	1	16	99	99	100	99
NC_017763.1	NC_007795.1	1561785	1562718	53.59	1961777	1963027	6.06	57	1	16	99	97	99	97
NZ_CP007659.1	NC_002951.2	1568275	1576904	45.31	358442	369368	2.81	15	1	31	99	99	100	99
NZ_CP007659.1	NC_002951.2	1568948	1576904	46.77	358442	369170	2.78	8	1	29	99	99	100	99
NZ_CP007659.1	NC_002951.2	1575976	1576904	53.62	358442	359696	5.56	8	1	15	100	99	100	98
