# Supplementary material for: Where did you come from, where did you go: Refining metagenomic analysis tools for horizontal gene transfer characterisation
Source: PLoS Comput Biol. 2019 Jul 23;15(7):e1007208. doi: 10.1371/journal.pcbi.1007208 (PMC6677323; doi:10.1371/journal.pcbi.1007208)
Supplement: S24 Table — (PDF) [file pcbi.1007208.s024.pdf]

S24 Table: Acceptor and donor candidates for ERR103394 run with yara, species filter and no samflag filter. Sampling sensitivity = 85. No taxon blacklist. No parent blacklist. No species blacklist. (-)0.000^* represents absolute values $< 0.000\bar{4}$. The supposed acceptor is marked in bold.

Candidate			MicrobeGPS metrics			DaisyGPS metrics	
Type	Name	Accession.Version	Number Reads	Validity	Heterogeneity	Donor Score	Acceptor Score
**Acceptor**	**Staphylococcus aureus subsp. aureus HO 5096 0412**	**NC_017763.1**	**183503**	**0.807**	**0.048**	**0.759**	**0.040**
Acceptor	Staphylococcus aureus subsp. aureus	NZ_CP007659.1	183292	0.801	0.05	0.751	0.04
Donor	Staphylococcus warneri SG1	NC_020164.1	250	0.004	0.656	-0.653	-0.000*
Donor	Staphylococcus pseudintermedius HKU10-03	NC_014925.1	747	0.001	0.584	-0.582	-0.000*
Donor	Staphylococcus epidermidis RP62A	NC_002976.3	2358	0.005	0.546	-0.541	-0.000*
Donor	Staphylococcus haemolyticus JCSC1435	NC_007168.1	1541	0.005	0.301	-0.296	-0.000*
Donor	Staphylococcus aureus subsp. aureus COL	NC_002951.2	20650	0.100	0.246	-0.146	-0.001
Acceptor-like Donor	Staphylococcus aureus subsp. aureus DSM 20231	NZ_CP011526.1	16141	0.102	0.091	0.011	0.000*
