# Supplementary material for: Where did you come from, where did you go: Refining metagenomic analysis tools for horizontal gene transfer characterisation
Source: PLoS Comput Biol. 2019 Jul 23;15(7):e1007208. doi: 10.1371/journal.pcbi.1007208 (PMC6677323; doi:10.1371/journal.pcbi.1007208)
Supplement: S25 Table — (PDF) [file pcbi.1007208.s025.pdf]

S25 Table: Results for ERR103394 run with yara, gustaf, species filter and no samflag filter. Sampling sensitivity = 90. Split read threshold = 3. No taxon blacklist. No parent blacklist. No species blacklist.

Organism		Acceptor			Donor			Read Evidence			Evidence Filter			
Acceptor	Donor	Start	End	Coverage	Start	End	Coverage	Split	Spanning	Within	A-Cov	D-Cov	Spanning	Within
NZ_CP007659.1	NC_014925.1	36953	37046	319.63	906200	906301	89.36	6	17	18	100	100	100	100
NZ_CP007659.1	NC_014925.1	36953	37133	263.84	906200	906387	137.22	13	20	20	100	100	100	100
NZ_CP007659.1	NC_014925.1	36953	37152	254.4	906200	906409	135.31	9	19	20	100	100	100	100
NZ_CP007659.1	NC_014925.1	36999	37133	225.07	906256	906387	169.08	11	18	20	100	100	100	100
NZ_CP007659.1	NC_014925.1	36999	37152	217.61	906256	906409	161.88	7	18	20	100	100	100	100
NZ_CP007659.1	NC_014925.1	37045	37152	197.65	906300	906409	178.28	5	12	19	100	100	100	100
NC_017763.1	NC_002951.2	1554089	1562718	54.38	358442	369368	2.46	16	3	25	99	98	100	95
NC_017763.1	NC_002951.2	1554762	1562718	55.43	358442	369170	2.46	29	3	25	99	99	100	97
NC_017763.1	NC_014925.1	36952	37045	299.67	906200	906301	76.57	7	15	19	100	100	100	100
NC_017763.1	NC_014925.1	36952	37132	228.17	906200	906387	109.47	21	17	21	100	100	100	100
NC_017763.1	NC_014925.1	36952	37151	217.36	906200	906409	105.63	15	16	21	100	100	100	100
NC_017763.1	NC_014925.1	36998	37132	183.81	906256	906387	134.34	11	16	21	99	100	100	100
NC_017763.1	NC_014925.1	36998	37151	175.26	906256	906409	125.52	8	16	21	100	100	100	100
NC_017763.1	NC_014925.1	37044	37151	145.74	906300	906409	132.93	6	10	20	100	100	100	100
NZ_CP007659.1	NZ_CP011526.1	1568903	1575973	55.17	846399	854374	10.79	13	3	234	97	99	100	99
NZ_CP007659.1	NZ_CP011526.1	1568903	1579178	54.74	842251	854374	19.62	5	3	747	98	100	99	100
NZ_CP007659.1	NZ_CP011526.1	1568953	1575973	55.21	846399	854250	10.95	26	3	234	99	98	100	98
NZ_CP007659.1	NZ_CP011526.1	1568953	1579178	54.76	842251	854250	19.82	10	3	747	100	100	100	100
NC_017763.1	NZ_CP011526.1	1554717	1561787	55.17	846399	854374	10.79	13	3	234	99	100	100	100
NC_017763.1	NZ_CP011526.1	1554717	1564992	54.74	842251	854374	19.62	5	3	747	99	100	100	100
NC_017763.1	NZ_CP011526.1	1554767	1561787	55.21	846399	854250	10.95	26	3	234	99	100	100	100
NC_017763.1	NZ_CP011526.1	1554767	1564992	54.76	842251	854250	19.82	10	3	747	97	100	100	100
NZ_CP007659.1	NC_002951.2	1568275	1576904	54.38	358442	369368	2.46	16	3	25	99	98	100	96
NZ_CP007659.1	NC_002951.2	1568948	1576904	55.43	358442	369170	2.46	29	3	25	99	99	100	97
