# Supplementary material for: Where did you come from, where did you go: Refining metagenomic analysis tools for horizontal gene transfer characterisation
Source: PLoS Comput Biol. 2019 Jul 23;15(7):e1007208. doi: 10.1371/journal.pcbi.1007208 (PMC6677323; doi:10.1371/journal.pcbi.1007208)
Supplement: S26 Table — (PDF) [file pcbi.1007208.s026.pdf]

S26 Table: Acceptor and donor candidates for ERR103395 run with yara, species filter and no samflag filter. Sampling sensitivity = 85. No taxon blacklist. No parent blacklist. No species blacklist. (-)0.000^* represents absolute values $< 0.000\bar{4}$.

Candidate			MicrobeGPS metrics			DaisyGPS metrics	
Type	Name	Accession.Version	Number Reads	Validity	Heterogeneity	Donor Score	Acceptor Score
Acceptor	Staphylococcus aureus subsp. aureus ECT-R 2	NC_017343.1	120322	0.591	0.070	0.521	0.013
Acceptor	Staphylococcus aureus subsp. aureus N315	NC_002745.2	121110	0.576	0.069	0.507	0.013
Donor	Enterococcus faecium Aus0004	NC_017022.1	471	0.001	0.974	-0.973	-0.000*
Donor	Staphylococcus epidermidis ATCC 12228	NC_004461.1	391	0.001	0.971	-0.97	-0.000*
Donor	Staphylococcus pseudintermedius HKU10-03	NC_014925.1	470	0.001	0.806	-0.805	-0.000*
Donor	Staphylococcus lugdunensis HKU09-01	NC_013893.1	59	0.003	0.765	-0.762	-0.000*
Donor	Staphylococcus warneri SG1	NC_020164.1	294	0.011	0.693	-0.683	-0.000*
Donor	Staphylococcus haemolyticus JCSC1435	NC_007168.1	362	0.002	0.556	-0.554	-0.000*
Acceptor-like Donor	Staphylococcus aureus subsp. aureus	NZ_CP009554.1	14824	0.093	0.091	0.002	0.000*
