# Supplementary material for: Where did you come from, where did you go: Refining metagenomic analysis tools for horizontal gene transfer characterisation
Source: PLoS Comput Biol. 2019 Jul 23;15(7):e1007208. doi: 10.1371/journal.pcbi.1007208 (PMC6677323; doi:10.1371/journal.pcbi.1007208)
Supplement: S27 Table — (PDF) [file pcbi.1007208.s027.pdf]

S27 Table: Results for ERR103395 run with yara, gustaf, species filter and no samflag filter. Sampling sensitivity = 90. Split read threshold = 3. No taxon blacklist. No parent blacklist. No species blacklist.

Organism		Acceptor			Donor			Read Evidence			Evidence Filter			
Acceptor	Donor	Start	End	Coverage	Start	End	Coverage	Split	Spanning	Within	A-Cov	D-Cov	Spanning	Within
NC_002745.2	NC_013893.1	2060607	2069048	16.44	2073055	2083555	11.08	28	8	339	1	100	100	100
NC_002745.2	NC_013893.1	2060607	2069067	16.47	2073055	2083576	11.07	18	6	339	2	100	99	100
NC_002745.2	NC_013893.1	2060762	2069048	16.74	2073192	2083555	11.11	7	8	339	1	100	100	100
NC_002745.2	NZ_CP009554.1	1142176	1142913	0.26	685582	686374	22.86	8	11	52	3	100	100	100
NC_002745.2	NZ_CP009554.1	1142176	1142913	0.26	685582	717267	0.66	12	14	53	5	94	97	94
NC_002745.2	NZ_CP009554.1	1142912	1142913	1.0	685581	716475	0.66	11	12	53	4	93	98	94
NC_002745.2	NZ_CP009554.1	2056699	2058174	0.02	2150234	2162636	2.65	10	6	43	2	98	99	94
NC_002745.2	NZ_CP009554.1	2056699	2060475	5.31	2150276	2162636	2.66	13	6	43	1	100	99	99
NC_002745.2	NZ_CP009554.1	2056699	2069076	10.31	2158985	2162636	3.14	24	34	8	2	99	100	90
NC_002745.2	NZ_CP009554.1	2058173	2069076	11.7	2150233	2158985	2.44	51	5	34	0	97	100	97
NC_002745.2	NZ_CP009554.1	2058173	2069105	11.7	2150233	2159011	2.48	7	5	34	3	100	100	98
NC_002745.2	NZ_CP009554.1	2058173	2069324	11.5	2150233	2159202	3.5	27	26	43	0	100	100	99
NC_002745.2	NZ_CP009554.1	2058173	2069355	11.57	2150233	2159253	3.62	7	26	43	0	100	100	96
NC_002745.2	NZ_CP009554.1	2060474	2069076	12.5	2150275	2158985	2.45	52	5	34	0	99	100	99
NC_002745.2	NZ_CP009554.1	2060474	2069105	12.5	2150275	2159011	2.49	8	5	34	2	100	100	98
NC_002745.2	NZ_CP009554.1	2060474	2069324	12.23	2150275	2159202	3.51	28	26	43	5	98	100	97
NC_002745.2	NZ_CP009554.1	2060474	2069355	12.31	2150275	2159253	3.63	8	26	43	1	100	100	99
NC_002745.2	NZ_CP009554.1	2060607	2065052	19.41	364874	369569	10.13	14	5	133	2	100	100	100
NC_002745.2	NZ_CP009554.1	2060607	2068738	12.04	361186	369569	7.19	18	1	154	0	100	100	100
NC_002745.2	NZ_CP009554.1	2065051	2068738	3.16	361186	364873	3.44	9	3	21	3	98	99	97
NC_002745.2	NZ_CP009554.1	2069075	2069324	2.76	2158984	2159202	45.94	89	32	8	4	100	100	100
NC_002745.2	NZ_CP009554.1	2069075	2069355	6.49	2158984	2159253	41.82	9	38	8	2	100	100	99
NC_002745.2	NZ_CP009554.1	2069104	2069324	1.65	2159010	2159202	50.14	12	32	8	3	100	100	99
NC_017343.1	NZ_CP009554.1	1100697	1101434	0.42	685582	686374	22.86	8	11	52	1	100	100	100
NC_017343.1	NZ_CP009554.1	1100697	1101434	0.42	685582	717267	0.66	15	14	53	1	99	99	98
NC_017343.1	NZ_CP009554.1	1101433	1101434	1.0	685581	716475	0.66	11	12	53	0	98	96	96
NC_002745.2	NC_004461.1	61651	61779	4.23	37793	55322	7.62	30	73	392	4	100	99	100
NC_002745.2	NC_004461.1	61651	61799	3.8	37814	55322	7.62	14	73	392	4	100	100	100
NC_002745.2	NC_004461.1	61651	61851	2.83	37866	55322	7.61	18	73	392	2	100	99	100
NC_002745.2	NC_004461.1	61755	61779	3.04	37793	55383	7.59	12	73	392	2	100	100	100
NC_002745.2	NC_004461.1	61755	61799	2.14	37814	55383	7.59	8	73	392	5	100	99	100
NC_002745.2	NC_004461.1	61755	61851	1.01	37866	55383	7.58	9	73	392	3	100	100	100
NC_002745.2	NC_004461.1	61778	62058	2.57	37792	57274	6.86	7	73	392	1	100	100	100
NC_002745.2	NC_004461.1	61778	62354	7.14	37792	57575	6.75	7	68	392	2	100	100	100
NC_002745.2	NC_004461.1	61778	62414	7.02	37792	57608	6.74	8	64	392	1	100	99	100
NC_002745.2	NC_004461.1	61798	62058	2.68	37813	57274	6.85	3	73	392	5	100	100	100
NC_002745.2	NC_004461.1	61798	62354	7.35	37813	57575	6.75	3	68	392	1	100	99	100
NC_002745.2	NC_004461.1	61798	62414	7.21	37813	57608	6.74	4	64	392	1	100	100	100
NC_002745.2	NC_004461.1	61850	62058	3.34	37865	57274	6.84	4	73	392	5	100	99	100
NC_002745.2	NC_004461.1	61850	62354	8.11	37865	57575	6.74	4	68	392	4	100	100	100
NC_002745.2	NC_004461.1	61850	62414	7.87	37865	57608	6.73	7	64	392	2	100	100	100
