# Supplementary material for: Where did you come from, where did you go: Refining metagenomic analysis tools for horizontal gene transfer characterisation
Source: PLoS Comput Biol. 2019 Jul 23;15(7):e1007208. doi: 10.1371/journal.pcbi.1007208 (PMC6677323; doi:10.1371/journal.pcbi.1007208)
Supplement: S28 Table — (PDF) [file pcbi.1007208.s028.pdf]

S28 Table: Acceptor and donor candidates for ERR103396 run with yara, species filter and no samflag filter. Sampling sensitivity = 85. No taxon blacklist. No parent blacklist. No species blacklist. (-)0.000^* represents absolute values $< 0.000\bar{4}$. The supposed acceptor is marked in bold.

Candidate			MicrobeGPS metrics			DaisyGPS metrics	
Type	Name	Accession.Version	Number Reads	Validity	Heterogeneity	Donor Score	Acceptor Score
**Acceptor**	**Staphylococcus aureus subsp. aureus HO 5096 0412**	**NC_017763.1**	**222016**	**0.817**	**0.042**	**0.775**	**0.043**
Acceptor	Staphylococcus aureus subsp. aureus	NZ_CP007659.1	223952	0.815	0.049	0.767	0.043
Donor	Staphylococcus pseudintermedius ED99	NC_017568.1	536	0.002	0.708	-0.707	-0.000*
Donor	Staphylococcus warneri SG1	NC_020164.1	267	0.003	0.696	-0.693	-0.000*
Donor	Staphylococcus epidermidis RP62A	NC_002976.3	1067	0.003	0.582	-0.579	-0.000*
Donor	Staphylococcus haemolyticus JCSC1435	NC_007168.1	370	0.003	0.492	-0.489	-0.000*
Donor	Staphylococcus aureus subsp. aureus COL	NC_002951.2	21752	0.098	0.156	-0.058	-0.000*
Acceptor-like Donor	Staphylococcus aureus subsp. aureus	NZ_CP012012.1	21332	0.097	0.094	0.003	0.000*
