# Supplementary material for: Where did you come from, where did you go: Refining metagenomic analysis tools for horizontal gene transfer characterisation
Source: PLoS Comput Biol. 2019 Jul 23;15(7):e1007208. doi: 10.1371/journal.pcbi.1007208 (PMC6677323; doi:10.1371/journal.pcbi.1007208)
Supplement: S29 Table — (PDF) [file pcbi.1007208.s029.pdf]

S29 Table: Results for ERR103396 run with yara, gustaf, species filter and no samflag filter. Sampling sensitivity = 90. Split read threshold = 3. No taxon blacklist. No parent blacklist. No species blacklist.

Organism		Acceptor			Donor			Read Evidence			Evidence Filter			
Acceptor	Donor	Start	End	Coverage	Start	End	Coverage	Split	Spanning	Within	A-Cov	D-Cov	Spanning	Within
NC_017763.1	NZ_CP012012.1	98589	98635	95.67	125862	126004	35.02	3	20	5	100	100	100	100
NC_017763.1	NC_017568.1	409730	409775	16.98	2481624	2485653	3.95	14	5	35	1	100	100	100
