# Supplementary material for: Where did you come from, where did you go: Refining metagenomic analysis tools for horizontal gene transfer characterisation
Source: PLoS Comput Biol. 2019 Jul 23;15(7):e1007208. doi: 10.1371/journal.pcbi.1007208 (PMC6677323; doi:10.1371/journal.pcbi.1007208)
Supplement: S30 Table — (PDF) [file pcbi.1007208.s030.pdf]

S30 Table: Acceptor and donor candidates for ERR103397 run with yara, species filter and no samflag filter. Sampling sensitivity = 85. No taxon blacklist. No parent blacklist. No species blacklist. (-)0.000^* represents absolute values $< 0.000\bar{4}$.

**Candidate**			**MicrobeGPS metrics**			**DaisyGPS metrics**	
Type	Name	Accession.Version	Number Reads	Validity	Heterogeneity	Donor Score	Acceptor Score
Acceptor	Staphylococcus aureus subsp. aureus MSSA476	NC_002953.3	84971	0.634	0.094	0.540	0.017
Acceptor	Staphylococcus aureus subsp. aureus MW2	NC_003923.1	83556	0.621	0.089	0.531	0.017
Donor	Staphylococcus pseudintermedius HKU10-03	NC_014925.1	3645	0.002	0.744	-0.742	-0.001
Donor	Staphylococcus warneri SG1	NC_020164.1	168	0.003	0.69	-0.697	-0.000*
Donor	Staphylococcus haemolyticus JCSC1435	NC_007168.1	2650	0.004	0.604	-0.600	-0.001
Donor	Staphylococcus epidermidis RP62A	NC_002976.3	1082	0.002	0.583	-0.581	-0.000*
Donor	Staphylococcus lugdunensis HKU09-01	NC_013893.1	3709	0.004	0.356	-0.352	-0.001
Donor	Staphylococcus aureus subsp. aureus	NZ_CP009554.1	19819	0.092	0.314	-0.222	-0.002
Acceptor-like Donor	Staphylococcus aureus subsp. aureus	NZ_CP009361.1	9253	0.097	0.092	0.005	0.000*
