# Supplementary material for: Where did you come from, where did you go: Refining metagenomic analysis tools for horizontal gene transfer characterisation
Source: PLoS Comput Biol. 2019 Jul 23;15(7):e1007208. doi: 10.1371/journal.pcbi.1007208 (PMC6677323; doi:10.1371/journal.pcbi.1007208)
Supplement: S31 Table — (PDF) [file pcbi.1007208.s031.pdf]

S31 Table: Results for ERR103397 run with yara, gustaf, species filter and no samflag filter. Sampling sensitivity = 90. Split read threshold = 3. No taxon blacklist. No parent blacklist. No species blacklist.

Organism		Acceptor			Donor			Read Evidence			Evidence Filter			
Acceptor	Donor	Start	End	Coverage	Start	End	Coverage	Split	Spanning	Within	A-Cov	D-Cov	Spanning	Within
NC_003923.1	NC_007168.1	44986	45306	40.24	66689	67028	5.47	6	1	2	98	100	100	100
NC_003923.1	NC_002976.3	44776	44988	12.98	2520640	2520803	10.01	4	4	1	6	100	100	100
NC_003923.1	NC_002976.3	44987	45380	36.37	2520639	2561294	0.64	14	58	46	96	95	98	95
NC_003923.1	NC_002976.3	44987	45606	31.5	2520639	2561094	0.63	4	58	44	95	96	100	97
NC_003923.1	NC_002976.3	45026	45380	37.73	2561294	2561636	10.28	14	3	3	100	99	100	99
NC_003923.1	NC_002976.3	45026	45606	32.0	2561094	2561636	7.1	4	3	4	92	98	100	98
NC_003923.1	NC_002976.3	45026	45870	27.86	2560793	2561636	6.17	6	4	4	91	99	100	100
NC_003923.1	NC_002976.3	45070	45307	38.1	2561337	2561580	12.23	4	5	3	94	100	100	100
NC_003923.1	NC_002976.3	45070	45380	38.28	2561294	2561580	12.12	40	5	3	98	100	100	100
NC_002953.3	NC_007168.1	41508	57483	26.01	67036	120082	2.7	20	4	471	94	98	95	98
