# Supplementary material for: Where did you come from, where did you go: Refining metagenomic analysis tools for horizontal gene transfer characterisation
Source: PLoS Comput Biol. 2019 Jul 23;15(7):e1007208. doi: 10.1371/journal.pcbi.1007208 (PMC6677323; doi:10.1371/journal.pcbi.1007208)
Supplement: S32 Table — (PDF) [file pcbi.1007208.s032.pdf]

S32 Table: Acceptor and donor candidates for ERR103398 run with yara, species filter and no samflag filter. Sampling sensitivity = 85. No taxon blacklist. No parent blacklist. No species blacklist. (-)0.000^* represents absolute values $< 0.000\bar{4}$.

Candidate			MicrobeGPS metrics			DaisyGPS metrics	
Type	Name	Accession.Version	Number Reads	Validity	Heterogeneity	Donor Score	Acceptor Score
Acceptor	Staphylococcus aureus subsp. aureus MSSA476	NC_002953.3	192949	0.671	0.11	0.562	0.017
Acceptor	Staphylococcus aureus subsp. aureus MW2	NC_003923.1	189418	0.658	0.103	0.555	0.016
Donor	Staphylococcus pseudintermedius HKU10-03	NC_014925.1	16866	0.002	0.745	-0.742	-0.002
Donor	Staphylococcus warneri SG1	NC_020164.1	461	0.003	0.69	-0.697	-0.000*
Donor	Staphylococcus epidermidis PM221	NZ_HG813242.1	4779	0.001	0.656	-0.655	-0.001
Donor	Staphylococcus haemolyticus JCSC1435	NC_007168.1	12023	0.004	0.636	-0.632	-0.001
Donor	Staphylococcus lugdunensis HKU09-01	NC_013893.1	16800	0.004	0.356	-0.351	-0.001
Donor	Staphylococcus aureus subsp. aureus	NZ_CP009554.1	70966	0.095	0.398	-0.304	-0.003
Acceptor-like Donor	Staphylococcus aureus CA-347	NC_021554.1	18666	0.098	0.090	0.007	0.000*
