# Supplementary material for: Where did you come from, where did you go: Refining metagenomic analysis tools for horizontal gene transfer characterisation
Source: PLoS Comput Biol. 2019 Jul 23;15(7):e1007208. doi: 10.1371/journal.pcbi.1007208 (PMC6677323; doi:10.1371/journal.pcbi.1007208)
Supplement: S33 Table — (PDF) [file pcbi.1007208.s033.pdf]

S33 Table: Results for ERR103398 run with yara, gustaf, species filter and no samflag filter. Sampling sensitivity = 90. Split read threshold = 3. No taxon blacklist. No parent blacklist. No species blacklist.

Organism		Acceptor			Donor			Read Evidence			Evidence Filter			
Acceptor	Donor	Start	End	Coverage	Start	End	Coverage	Split	Spanning	Within	A-Cov	D-Cov	Spanning	Within
NC_002953.3	NC_007168.1	27843	41291	63.48	67243	97433	4.35	18	2	436	95	97	99	99
NC_002953.3	NC_007168.1	27843	41907	63.94	66699	97433	4.54	3	4	446	91	99	99	99
NC_002953.3	NC_007168.1	27843	57484	69.06	94688	97433	47.35	7	2	431	99	100	100	100
NC_002953.3	NC_007168.1	41290	41907	73.94	66699	67242	15.3	9	6	14	96	100	100	100
NC_002953.3	NC_007168.1	41290	57483	73.69	67242	120082	6.24	77	6	1105	99	94	99	97
NC_002953.3	NC_007168.1	41290	57484	73.69	30072	67242	0.28	25	7	16	100	93	100	97
NC_002953.3	NC_007168.1	41508	41605	95.51	66925	67036	6.53	17	6	1	97	100	100	100
NC_002953.3	NC_007168.1	41508	41907	90.78	66699	67036	7.76	5	1	2	98	100	100	100
NC_002953.3	NC_007168.1	41508	57483	74.11	67036	120082	6.32	39	10	1111	98	91	94	95
NC_002953.3	NC_007168.1	41508	57484	74.11	67036	94688	0.25	13	8	11	100	93	97	95
NC_002953.3	NC_007168.1	41604	41907	89.3	66699	66924	8.38	9	2	1	97	99	100	99
NC_002953.3	NC_007168.1	41604	57483	73.98	66924	120082	6.32	77	15	1112	100	92	99	94
NC_002953.3	NC_007168.1	41906	57483	73.68	66698	120082	6.33	21	15	1115	100	91	100	94
NC_002953.3	NC_007168.1	41906	57484	73.68	66698	94688	0.34	8	13	15	100	92	100	96
NC_002953.3	NZ_HG813242.1	34149	41829	68.51	35795	85587	4.98	78	19	287	99	99	97	99
NC_002953.3	NZ_HG813242.1	34149	41907	68.57	35721	85587	4.97	50	19	287	95	97	98	97
NC_002953.3	NZ_HG813242.1	34149	57484	71.98	54292	85587	6.1	34	3	280	97	97	97	97
NC_002953.3	NZ_HG813242.1	34149	57484	71.98	57395	85587	5.91	42	2	207	100	97	100	97
NC_002953.3	NZ_HG813242.1	34180	41829	68.6	35795	85647	4.97	240	19	287	93	96	97	96
NC_002953.3	NZ_HG813242.1	34180	41907	68.66	35721	85647	4.96	142	19	287	97	95	96	95
NC_002953.3	NZ_HG813242.1	34180	57484	72.02	54292	85647	6.09	86	3	280	100	96	100	96
NC_002953.3	NZ_HG813242.1	34180	57484	72.02	57395	85647	5.9	114	2	207	100	97	100	97
NC_002953.3	NZ_HG813242.1	41828	56986	72.4	35794	85689	4.98	81	19	287	99	94	97	94
NC_002953.3	NZ_HG813242.1	41828	57484	73.68	35794	57395	3.75	43	2	70	99	94	100	93
NC_002953.3	NZ_HG813242.1	41828	57484	73.68	35794	62757	9.18	15	3	286	100	97	97	97
NC_002953.3	NZ_HG813242.1	41906	56986	72.39	35720	85689	4.97	143	19	287	100	93	98	93
NC_002953.3	NZ_HG813242.1	41906	57484	73.68	35720	57395	3.74	67	2	70	100	98	98	98
NC_002953.3	NZ_HG813242.1	41906	57484	73.68	35720	62757	9.16	11	3	286	100	99	98	99
NC_002953.3	NZ_HG813242.1	56985	57484	112.78	54292	85688	6.1	64	3	280	100	97	98	97
NC_002953.3	NZ_HG813242.1	56985	57484	112.78	57395	85688	5.91	80	2	207	100	98	99	98
NC_003923.1	NC_007168.1	44606	45306	69.87	66689	67411	10.45	29	2	16	95	100	100	100
NC_003923.1	NC_007168.1	45026	45143	97.32	66870	66987	2.7	9	4	2	100	99	100	99
NC_003923.1	NC_007168.1	45026	45306	93.51	66689	66987	17.21	25	6	13	98	100	100	100
NC_003923.1	NC_007168.1	45062	45306	93.32	66689	66930	20.85	19	3	11	99	99	100	100
NC_003923.1	NC_007168.1	45082	45306	92.39	66689	66929	20.93	10	4	11	98	100	100	100
NC_003923.1	NC_007168.1	45142	45306	90.77	66689	66869	26.71	10	5	11	97	99	100	99
NC_002953.3	NC_021554.1	41508	41593	91.24	60998	61108	10.47	19	2	5	96	99	100	99
NC_002953.3	NC_021554.1	41508	41884	81.12	60998	61399	20.48	7	1	11	95	99	100	99
NC_002953.3	NC_021554.1	41548	41884	80.93	61045	61399	21.66	4	2	9	96	100	100	100
NC_002953.3	NC_021554.1	41592	41884	78.23	61107	61399	24.23	22	3	8	92	99	100	99
NC_003923.1	NC_021554.1	44986	45384	76.26	61006	61391	27.57	55	2	20	90	100	100	100
NC_003923.1	NC_021554.1	45026	45306	80.71	61045	61347	29.43	7	3	18	95	100	100	100
NC_003923.1	NC_021554.1	45026	45384	76.52	61045	61391	29.23	113	3	18	94	100	100	100
NC_003923.1	NC_021554.1	45062	45306	78.63	61102	61347	35.75	3	1	14	92	100	100	99
NC_003923.1	NC_021554.1	45062	45384	74.48	61102	61391	34.55	109	1	14	92	100	100	100
NC_003923.1	NC_021554.1	45082	45384	72.6	61105	61391	34.9	55	1	14	91	100	100	100
