# Supplementary material for: Where did you come from, where did you go: Refining metagenomic analysis tools for horizontal gene transfer characterisation
Source: PLoS Comput Biol. 2019 Jul 23;15(7):e1007208. doi: 10.1371/journal.pcbi.1007208 (PMC6677323; doi:10.1371/journal.pcbi.1007208)
Supplement: S34 Table — (PDF) [file pcbi.1007208.s034.pdf]

S34 Table: Acceptor and donor candidates for ERR159680 run with yara, species filter and no samflag filter. Sampling sensitivity = 85. No taxon blacklist. No parent blacklist. No species blacklist. (-)0.000^* represents absolute values $< 0.000\bar{4}$.

Candidate			MicrobeGPS metrics			DaisyGPS metrics	
Type	Name	Accession.Version	Number Reads	Validity	Heterogeneity	Donor Score	Acceptor Score
Acceptor	Staphylococcus aureus subsp. aureus MRSA252	NC_002952.2	236631	0.892	0.047	0.845	0.043
Acceptor	Staphylococcus aureus subsp. aureus	NZ_CP009554.1	227305	0.871	0.046	0.825	0.041
Donor	Staphylococcus pseudintermedius ED99	NC_017568.1	780	0.003	0.946	-0.944	-0.000*
Donor	Streptococcus pasteurianus ATCC 43144	NC_015600.1	397	0.001	0.828	-0.827	-0.000*
Donor	Staphylococcus epidermidis RP62A	NC_002976.3	6553	0.019	0.804	-0.785	-0.001
Donor	Streptococcus gallolyticus UCN34	NC_013798.1	453	0.001	0.752	-0.751	-0.000*
Donor	Staphylococcus haemolyticus JCSC1435	NC_007168.1	1295	0.005	0.516	-0.511	-0.000*
Donor	Staphylococcus lugdunensis HKU09-01	NC_013893.1	494	0.003	0.356	-0.353	-0.000*
Acceptor-like Donor	Staphylococcus aureus subsp. aureus	NZ_AP014652.1	17647	0.096	0.085	0.011	0.000*
