# Supplementary material for: Where did you come from, where did you go: Refining metagenomic analysis tools for horizontal gene transfer characterisation
Source: PLoS Comput Biol. 2019 Jul 23;15(7):e1007208. doi: 10.1371/journal.pcbi.1007208 (PMC6677323; doi:10.1371/journal.pcbi.1007208)
Supplement: S35 Table — (PDF) [file pcbi.1007208.s035.pdf]

S35 Table: Results for ERR159680 run with yara, gustaf, species filter and no samflag filter. Sampling sensitivity = 90. Split read threshold = 3. No taxon blacklist. No parent blacklist. No species blacklist.

Organism		Acceptor			Donor			Read Evidence			Evidence Filter			
Acceptor	Donor	Start	End	Coverage	Start	End	Coverage	Split	Spanning	Within	A-Cov	D-Cov	Spanning	Within
NZ_CP009554.1	NC_002976.3	34120	34123	12.67	2536574	2584194	38.98	30	19	5752	6	100	98	100
NZ_CP009554.1	NC_002976.3	859613	866305	63.76	1398260	1404973	0.3	27	4	5	100	98	100	98
NZ_CP009554.1	NC_013893.1	2130925	2133716	7.9	2343346	2345047	5.05	17	1	10	3	100	100	100
NZ_CP009554.1	NC_013893.1	2131388	2133716	4.04	2343670	2345047	6.23	16	1	10	0	100	100	100
NC_002952.2	NC_002976.3	906791	906792	12.0	1398259	1404972	0.3	18	4	5	1	94	100	97
NZ_CP009554.1	NZ_AP014652.1	414814	417301	46.9	438237	438358	11.31	4	2	5	96	99	100	99
NZ_CP009554.1	NZ_AP014652.1	2110903	2123964	16.67	2007772	2020977	17.83	6	4	762	0	98	99	98
NZ_CP009554.1	NZ_AP014652.1	2110903	2131317	11.83	2007772	2029781	21.15	3	4	1493	0	100	97	100
NZ_CP009554.1	NZ_AP014652.1	2110903	2134197	10.62	2007772	2030266	20.77	25	3	1493	0	99	99	99
NZ_CP009554.1	NZ_AP014652.1	2123963	2131021	1.86	2020976	2029466	27.01	5	1	729	0	98	100	98
NZ_CP009554.1	NZ_AP014652.1	2123963	2131317	3.23	2020976	2029781	26.14	7	2	731	1	98	100	98
NZ_CP009554.1	NZ_AP014652.1	2123963	2134197	2.91	2020976	2030266	24.94	51	1	731	0	100	99	100
NZ_CP009554.1	NZ_AP014652.1	2125253	2131317	1.84	2022297	2029781	30.59	3	3	731	0	98	100	98
NZ_CP009554.1	NZ_AP014652.1	2125253	2134197	1.92	2022297	2030266	28.92	25	2	731	0	99	100	99
NZ_CP009554.1	NZ_AP014652.1	2131020	2134197	5.24	2029465	2030266	2.97	25	1	2	2	97	100	97
NZ_CP009554.1	NZ_AP014652.1	2131316	2134197	2.09	2029780	2030266	3.19	49	6	2	1	97	100	96
NC_002952.2	NC_013893.1	413772	417366	53.37	2079996	2083590	0.78	5	4	2	100	99	100	100
