# Supplementary material for: Where did you come from, where did you go: Refining metagenomic analysis tools for horizontal gene transfer characterisation
Source: PLoS Comput Biol. 2019 Jul 23;15(7):e1007208. doi: 10.1371/journal.pcbi.1007208 (PMC6677323; doi:10.1371/journal.pcbi.1007208)
Supplement: S36 Table — (PDF) [file pcbi.1007208.s036.pdf]

S36 Table: Acceptor and donor candidates for ERR103400 run with yara, species filter and no samflag filter. Sampling sensitivity = 85. No taxon blacklist. No parent blacklist. No species blacklist. (-)0.000^* represents absolute values $< 0.000\bar{4}$. The supposed acceptor is marked in bold.

Candidate			MicrobeGPS metrics			DaisyGPS metrics	
Type	Name	Accession.Version	Number Reads	Validity	Heterogeneity	Donor Score	Acceptor Score
**Acceptor**	**Staphylococcus aureus subsp. aureus HO 5096 0412**	**NC_017763.1**	**484936**	**0.835**	**0.037**	**0.798**	**0.041**
Acceptor	Staphylococcus aureus subsp. aureus	NZ_CP007659.1	489699	0.832	0.048	0.784	0.041
Donor	Staphylococcus haemolyticus JCSC1435	NC_007168.1	3222	0.006	0.799	-0.792	-0.000*
Donor	Staphylococcus pseudintermedius ED99	NC_017568.1	1398	0.002	0.701	-0.699	-0.000*
Donor	Staphylococcus warneri SG1	NC_020164.1	583	0.003	0.695	-0.692	-0.000*
Donor	Staphylococcus epidermidis ATCC 12228	NC_004461.1	3245	0.005	0.483	-0.479	-0.000*
Donor	Staphylococcus lugdunensis HKU09-01	NC_013893.1	69	0.005	0.342	-0.337	-0.000*
Donor	Staphylococcus aureus subsp. aureus	NZ_CP009554.1	132861	0.21	0.254	-0.044	-0.001
Acceptor-like Donor	Staphylococcus aureus subsp. aureus T0131	NC_017347.1	50347	0.104	0.103	0.001	0.000*
