# Supplementary material for: Where did you come from, where did you go: Refining metagenomic analysis tools for horizontal gene transfer characterisation
Source: PLoS Comput Biol. 2019 Jul 23;15(7):e1007208. doi: 10.1371/journal.pcbi.1007208 (PMC6677323; doi:10.1371/journal.pcbi.1007208)
Supplement: S37 Table — (PDF) [file pcbi.1007208.s037.pdf]

S37 Table: Results for ERR103400 run with yara, gustaf, species filter and no samflag filter. Sampling sensitivity = 90. Split read threshold = 3. No taxon blacklist. No parent blacklist. No species blacklist.

Organism		Acceptor			Donor			Read Evidence			Evidence Filter			
Acceptor	Donor	Start	End	Coverage	Start	End	Coverage	Split	Spanning	Within	A-Cov	D-Cov	Spanning	Within
NZ_CP007659.1	NC_017347.1	36952	63749	105.32	2780055	2782476	52.24	24	43	358	92	99	100	99
NC_017763.1	NC_007168.1	44772	58518	112.86	67396	74115	52.21	29	1	1039	96	98	100	98
NC_017763.1	NC_007168.1	44772	58518	112.86	67396	74141	52.36	9	1	1045	94	99	100	99
NC_017763.1	NC_007168.1	44772	58661	113.09	67396	73961	51.35	79	1	1007	94	99	100	100
NC_017763.1	NC_007168.1	44772	58729	113.16	67396	73859	51.65	49	1	991	94	99	100	100
NC_017763.1	NC_007168.1	44772	58751	113.17	67396	73849	51.68	9	1	991	97	100	100	100
NC_017763.1	NC_007168.1	44772	63969	109.85	67396	68656	13.93	9	1	31	96	99	100	99
NC_017763.1	NC_007168.1	45010	58518	113.61	67122	74115	50.25	41	1	1040	96	99	100	99
NC_017763.1	NC_007168.1	45010	58518	113.61	67122	74141	50.39	13	1	1046	97	100	100	100
NC_017763.1	NC_007168.1	45010	58661	113.84	67122	73961	49.37	111	1	1008	96	99	100	100
NC_017763.1	NC_007168.1	45010	58729	113.91	67122	73859	49.63	69	1	992	95	99	100	100
NC_017763.1	NC_007168.1	45010	58751	113.91	67122	73849	49.66	13	1	992	97	100	100	100
NC_017763.1	NC_007168.1	45010	63969	110.35	67122	68656	11.81	13	1	32	92	99	100	99
NC_017763.1	NC_007168.1	45149	45440	132.36	66689	67061	16.42	94	4	5	95	100	100	100
NC_017763.1	NC_007168.1	45149	58518	114.17	67061	74115	49.88	22	1	1040	97	99	100	99
NC_017763.1	NC_007168.1	45149	58518	114.17	67061	74141	50.02	10	1	1046	98	100	100	100
NC_017763.1	NC_007168.1	45149	58661	114.4	67061	73961	49.0	52	1	1008	97	99	100	99
NC_017763.1	NC_007168.1	45149	58729	114.46	67061	73859	49.25	34	1	992	93	98	99	99
NC_017763.1	NC_007168.1	45149	58751	114.47	67061	73849	49.29	10	1	992	98	100	100	100
NC_017763.1	NC_007168.1	45149	63969	110.72	67061	68656	11.64	10	1	32	91	99	100	99
NC_017763.1	NC_007168.1	45439	58518	113.77	66688	74115	48.2	27	8	1045	94	99	99	100
NC_017763.1	NC_007168.1	45439	58518	113.77	66688	74141	48.35	7	8	1051	95	100	100	100
NC_017763.1	NC_007168.1	45439	58661	114.0	66688	73961	47.34	77	8	1013	97	100	99	100
NC_017763.1	NC_007168.1	45439	58729	114.07	66688	73859	47.55	47	8	997	97	99	100	99
NC_017763.1	NC_007168.1	45439	58751	114.07	66688	73849	47.58	7	8	997	97	96	100	96
NC_017763.1	NC_007168.1	45439	63969	110.38	66688	68656	12.57	7	8	37	94	100	100	100
NZ_CP007659.1	NC_004461.1	34160	34165	33.4	95612	110079	29.54	150	87	1321	0	100	97	100
NZ_CP007659.1	NC_004461.1	34160	36402	120.83	70358	110079	10.93	27	76	1321	95	100	99	100
NZ_CP007659.1	NC_004461.1	34164	36402	120.99	70358	95611	0.28	24	42	3	94	98	100	97
NZ_CP007659.1	NC_004461.1	44952	44985	50.7	37902	55503	0.48	4	2	6	5	94	99	94
NC_017763.1	NZ_CP009554.1	80759	82440	679.04	690422	696668	404.85	5	179	7590	100	100	100	100
NC_017763.1	NZ_CP009554.1	82439	82964	358.88	690421	696666	405.02	3	22	7591	99	100	99	100
NC_017763.1	NC_004461.1	34159	34164	33.4	95612	110079	29.54	150	87	1321	3	100	100	100
NC_017763.1	NC_004461.1	34159	36401	120.83	70358	110079	10.93	27	76	1321	95	100	98	100
NC_017763.1	NC_004461.1	34163	36401	120.99	70358	95611	0.28	24	42	3	95	99	99	95
NC_017763.1	NC_004461.1	44951	44984	50.7	37902	55503	0.48	4	2	6	5	96	100	92
NZ_CP007659.1	NC_007168.1	44773	58519	112.86	67396	74115	52.21	29	1	1039	96	99	98	100
NZ_CP007659.1	NC_007168.1	44773	58519	112.86	67396	74141	52.36	9	1	1045	97	99	100	99
NZ_CP007659.1	NC_007168.1	44773	58662	113.09	67396	73961	51.35	79	1	1007	97	100	100	100
NZ_CP007659.1	NC_007168.1	44773	58730	113.16	67396	73859	51.65	49	1	991	97	100	100	100
NZ_CP007659.1	NC_007168.1	44773	58752	113.17	67396	73849	51.68	9	1	991	98	100	100	100
NZ_CP007659.1	NC_007168.1	44773	63970	109.85	67396	68656	13.93	9	1	31	92	100	100	100
NZ_CP007659.1	NC_007168.1	45011	58519	113.61	67122	74115	50.25	41	1	1040	95	99	99	100
NZ_CP007659.1	NC_007168.1	45011	58519	113.61	67122	74141	50.39	13	1	1046	96	99	99	100
NZ_CP007659.1	NC_007168.1	45011	58662	113.84	67122	73961	49.37	111	1	1008	96	100	100	100
NZ_CP007659.1	NC_007168.1	45011	58730	113.91	67122	73859	49.63	69	1	992	90	99	100	99
NZ_CP007659.1	NC_007168.1	45011	58752	113.91	67122	73849	49.66	13	1	992	98	100	100	100
NZ_CP007659.1	NC_007168.1	45011	63970	110.35	67122	68656	11.81	13	1	32	98	97	100	99
NZ_CP007659.1	NC_007168.1	45150	45441	132.36	66689	67061	16.42	94	4	5	93	100	100	100
NZ_CP007659.1	NC_007168.1	45150	58519	114.17	67061	74115	49.88	22	1	1040	96	100	100	100
NZ_CP007659.1	NC_007168.1	45150	58519	114.17	67061	74141	50.02	10	1	1046	96	100	100	100
NZ_CP007659.1	NC_007168.1	45150	58662	114.4	67061	73961	49.0	52	1	1008	95	100	99	100
NZ_CP007659.1	NC_007168.1	45150	58730	114.46	67061	73859	49.25	34	1	992	99	99	100	99
NZ_CP007659.1	NC_007168.1	45150	58752	114.47	67061	73849	49.29	10	1	992	96	98	100	99
NZ_CP007659.1	NC_007168.1	45150	63970	110.72	67061	68656	11.64	10	1	32	91	100	100	100
NZ_CP007659.1	NC_007168.1	45440	58519	113.77	66688	74115	48.2	27	8	1045	98	100	100	100
NZ_CP007659.1	NC_007168.1	45440	58519	113.77	66688	74141	48.35	7	8	1051	96	100	100	99
NZ_CP007659.1	NC_007168.1	45440	58662	114.0	66688	73961	47.34	77	8	1013	99	100	100	100
NZ_CP007659.1	NC_007168.1	45440	58730	114.07	66688	73859	47.55	47	8	997	96	100	100	100
NZ_CP007659.1	NC_007168.1	45440	58752	114.07	66688	73849	47.58	7	8	997	94	100	100	100
NZ_CP007659.1	NC_007168.1	45440	63970	110.38	66688	68656	12.57	7	8	37	98	100	100	100
NC_017763.1	NC_017568.1	409726	409769	39.98	2481629	2485653	7.69	41	4	51	1	100	100	100
NC_017763.1	NC_017568.1	409747	409769	42.36	2481607	2485653	7.78	30	4	51	2	100	100	99
NZ_CP007659.1	NZ_CP009554.1	80760	82441	678.48	690422	696668	404.87	5	179	7590	100	100	100	100
