# Supplementary material for: Where did you come from, where did you go: Refining metagenomic analysis tools for horizontal gene transfer characterisation
Source: PLoS Comput Biol. 2019 Jul 23;15(7):e1007208. doi: 10.1371/journal.pcbi.1007208 (PMC6677323; doi:10.1371/journal.pcbi.1007208)
Supplement: S38 Table — (PDF) [file pcbi.1007208.s038.pdf]

S38 Table: Acceptor and donor candidates for ERR103402 run with yara, species filter and no samflag filter. Sampling sensitivity = 85. No taxon blacklist. No parent blacklist. No species blacklist. (-)0.000^* represents absolute values $< 0.000\bar{4}$. The supposed acceptor is marked in bold.

Candidate			MicrobeGPS metrics			DaisyGPS metrics	
Type	Name	Accession.Version	Number Reads	Validity	Heterogeneity	Donor Score	Acceptor Score
Acceptor	Staphylococcus aureus subsp. aureus	NZ_CP007659.1	169032	0.804	0.05	0.754	0.04
**Acceptor**	**Staphylococcus aureus subsp. aureus HO 5096 0412**	**NC_017763.1**	**167480**	**0.806**	**0.052**	**0.754**	**0.039**
Donor	Staphylococcus warneri SG1	NC_020164.1	231	0.003	0.69	-0.697	-0.000^*
Donor	Staphylococcus pseudintermedius ED99	NC_017568.1	1176	0.002	0.657	-0.655	-0.000^*
Donor	Staphylococcus epidermidis RP62A	NC_002976.3	786	0.003	0.578	-0.575	-0.000^*
Donor	Staphylococcus lugdunensis HKU09-01	NC_013893.1	676	0.001	0.357	-0.355	-0.000^*
Donor	Staphylococcus haemolyticus JCSC1435	NC_007168.1	1123	0.003	0.351	-0.348	-0.000^*
Donor	Staphylococcus aureus subsp. aureus str. JKD6008	NC_017341.1	18272	0.097	0.19	-0.103	-0.001
Acceptor-like Donor	Staphylococcus aureus subsp. aureus	NZ_CP009423.1	17888	0.096	0.085	0.011	0.000^*
