# Supplementary material for: Where did you come from, where did you go: Refining metagenomic analysis tools for horizontal gene transfer characterisation
Source: PLoS Comput Biol. 2019 Jul 23;15(7):e1007208. doi: 10.1371/journal.pcbi.1007208 (PMC6677323; doi:10.1371/journal.pcbi.1007208)
Supplement: S39 Table — (PDF) [file pcbi.1007208.s039.pdf]

S39 Table: Results for ERR103402 run with yara, gustaf, species filter and no samflag filter. Sampling sensitivity = 90. Split read threshold = 3. No taxon blacklist. No parent blacklist. No species blacklist.

Organism		Acceptor			Donor			Read Evidence			Evidence Filter			
Acceptor	Donor	Start	End	Coverage	Start	End	Coverage	Split	Spanning	Within	A-Cov	D-Cov	Spanning	Within
NZ_CP007659.1	NC_020164.1	2038921	2038922	83.0	121511	123832	2.22	48	24	12	100	100	100	100
NC_017763.1	NC_020164.1	2024903	2024904	83.0	121511	123832	2.22	52	24	12	99	100	99	100
NZ_CP007659.1	NC_017341.1	2036785	2038062	62.16	2760130	2761402	144.91	6	48	540	99	100	100	100
NZ_CP007659.1	NC_013893.1	2036709	2038062	66.05	1722127	1723472	67.35	10	10	2	100	100	100	100
NZ_CP007659.1	NC_013893.1	2036785	2038063	62.02	949399	950670	74.87	9	1	3	99	100	100	100
NZ_CP007659.1	NC_013893.1	2036785	2038062	62.03	1722127	1723395	70.11	18	51	2	100	100	100	100
NC_017763.1	NC_013893.1	2022691	2024044	66.05	1722127	1723472	67.46	10	10	2	100	100	100	100
NC_017763.1	NC_013893.1	2022767	2024045	62.02	949399	950670	74.75	9	1	3	99	100	100	100
NC_017763.1	NC_013893.1	2022767	2024044	62.03	1722127	1723395	70.23	18	51	2	100	100	100	100
NC_017763.1	NC_017341.1	2022767	2024044	62.16	2760130	2761402	144.91	6	48	540	99	100	100	100
NC_017763.1	NC_007168.1	2022767	2024044	62.16	1828214	1829486	144.91	10	48	540	99	100	100	100
NZ_CP007659.1	NC_007168.1	2036785	2038062	62.16	1828214	1829486	144.91	10	48	540	100	100	100	100
